# The Therapeutic Efficacy and Mechanism of Action of Gnetin C, a Natural Compound from the Melinjo Plant, in a Preclinical Mouse Model of Advanced Prostate Cancer

**DOI:** 10.3390/cancers16071344

**Published:** 2024-03-29

**Authors:** Gisella Campanelli, Ekniel Francois, Prashanth Parupathi, Lakshmi Sirisha Devarakonda, Ching Yang, Avinash Kumar, Anait S. Levenson

**Affiliations:** 1 Department of Veterinary Biomedical Sciences, College of Veterinary Medicine, Long Island University, Brookville, NY 11548, USA; gisella.campanelli@liu.edu (G.C.); ching.yang@liu.edu (C.Y.); 2 Division of Pharmaceutical Sciences, Arnold & Marie Schwartz College of Pharmacy and Health Sciences, Long Island University, Brooklyn, NY 11201, USA; ekniel.francois@my.liu.edu (E.F.); prashanth.parupathi@my.liu.edu (P.P.); lakshmisirisha.devarakonda@my.liu.edu (L.S.D.)

**Keywords:** natural products, plant-derived polyphenols, gnetin C, targeted therapeutics, anticancer effects, MTA1/mTOR, advanced prostate cancer

## Abstract

**Simple Summary:**

Incidence and mortality rates for prostate cancer remain high due to advanced disease characterized by the heterogeneous activation of numerous molecular pathways. In the current study, utilizing a genetic mouse model of advanced prostate cancer with hyperactivated metastasis-associated protein 1/mammalian target of rapamycin (MTA1/mTOR) tumor-promoting pathway, we show for the first time that gnetin C, a natural compound from the melinjo plant, blocks the progression of prostate cancer by reducing cell proliferation and angiogenesis and promoting cell death through the efficient targeting of the MTA1/mTOR pathway. These data may provide a foundation from which to explore gnetin C as a monotherapy and/or combination therapy with approved drugs against advanced prostate cancer in patients with a loss of phosphatase and tensin homolog (PTEN) expression and activated MTA1/mTOR signaling.

**Abstract:**

The metastasis-associated protein 1/protein kinase B (MTA1/AKT) signaling pathway has been shown to cooperate in promoting prostate tumor growth. Targeted interception strategies by plant-based polyphenols, specifically stilbenes, have shown great promise against MTA1-mediated prostate cancer progression. In this study, we employed a prostate-specific transgenic mouse model with MTA1 overexpression on the background of phosphatase and tensin homolog (*Pten*) null (*R26^MTA1^*; *Pten^f/f^*) and PC3M prostate cancer cells which recapitulate altered molecular pathways in advanced prostate cancer. Mechanistically, the MTA1 knockdown or pharmacological inhibition of MTA1 by gnetin C (dimer resveratrol) in cultured PC3M cells resulted in the marked inactivation of mammalian target of rapamycin (mTOR) signaling. In vivo, mice tolerated a daily intraperitoneal treatment of gnetin C (7 mg/kg bw) for 12 weeks without any sign of toxicity. Treatment with gnetin C markedly reduced cell proliferation and angiogenesis and promoted apoptosis in mice with advanced prostate cancer. Further, in addition to decreasing MTA1 levels in prostate epithelial cells, gnetin C significantly reduced mTOR signaling activity in prostate tissues, including the activity of mTOR-target proteins: p70 ribosomal protein S6 kinase (S6K) and eukaryotic translational initiation factor 4E (elF4E)-binding protein 1 (4EBP1). Collectively, these findings established gnetin C as a new natural compound with anticancer properties against MTA1/AKT/mTOR-activated prostate cancer, with potential as monotherapy and as a possible adjunct to clinically approved mTOR pathway inhibitors in the future.

## 1. Introduction

Prostate cancer patients are a heterogeneous group with a prognosis ranging from full recovery to malignant and lethal disease. Between 2007 and 2014, the number of prostate cancers diagnosed declined due to changes in screening recommendations concerning the detection of prostate-specific antigen. Consequently, the incidence rate for advanced stage prostate cancer has increased by about 5% per year [1]. Therefore, an urgent need exists to better understand the biological basis for the progression to aggressive disease and metastasis. Prostate tumors begin from preinvasive lesions such as prostatic intraepithelial neoplasia (PIN), which eventually progress to adenocarcinoma and, in some cases, metastatic disease. Different phases of prostate cancer are characterized by a distinct molecular makeup, during which various signaling pathways are activated. While androgen receptor (AR) signaling is considered the most critical pathway in hormone-sensitive disease, hormone-refractory tumors, which emerge after failing hormone deprivation therapy, are governed by the activation of other tumor-promoting networks, such as the phosphatase and tensin homolog (PTEN) loss of function-associated activation of the protein kinase B/mammalian target of rapamycin (AKT/mTOR) cell survival signaling pathway [2,3,4,5,6,7]. Yet another pathway having major implications in advanced prostate cancer is metastasis-associated protein 1 (MTA1) signaling, which is strongly associated with clinically aggressive prostate cancer [8,9]. We have previously reported increased levels of MTA1 in *Pten*-deficient and *Pten*-loss mouse models of prostate cancer, which affected downstream inflammation and cell survival pathways [10]. Our initial finding that reduced levels of MTA1 were inversely correlated with PTEN acetylation and activation, which resulted in significantly inhibited disease progression partly through the inhibition of p-AKT, revealed a novel deregulated signaling network in prostate cancer: the MTA1/PTEN/AKT pathway [10,11]. Furthermore, our recent studies in a mouse model of premalignant high-risk prostate cancer (*R26^MTA^*; *Pten^+/f^*; *Pb-Cre^+^*) demonstrated that targeting the MTA1/PTEN/AKT signaling pathway by diets supplemented with natural stilbenes, such as pterostilbene and gnetin C (dimer resveratrol), was effective in preventing prostate cancer progression from high-grade PIN to adenocarcinoma [12,13].

Despite significant advances in developing pharmacological inhibitors of PI3K/AKT/mTOR kinases, the results from clinical trials in prostate cancer demonstrated severe toxicity [14] and a lack of clinical benefit [15]. In the last two decades, there has been much interest in the potential health benefits of plant-based natural polyphenols as anti-inflammatory and anticancer agents [16,17,18]. Different classes of polyphenols have been shown to have anticancer effects through numerous signaling pathways including PI3K/AKT/mTOR [19,20,21]. Our group systematically reported on MTA1-mediated anticancer effects by members of the stilbene family of polyphenols, namely resveratrol, pterostilbene, and gnetin C [10,22]. We recently demonstrated gnetin C as a preclinical tumor growth inhibitor in prostate cancer xenografts and as a dietary interceptive agent in a transgenic mouse model of prostate cancer [13,23]. Based on our previous findings, we hypothesized that gnetin C might be effective as an MTA1-targeted therapeutic agent against advanced prostate cancer. Two major advantages will be gained: (1) new biomarkers such as MTA1 involved in the AKT/mTOR pathway may allow for the selection of a subpopulation of patients more likely to benefit from inhibitors of specific pathways that do not alter kinase activity; (2) safe natural compounds with the ability to inhibit this pathway through MTA1 may provide the rationale for novel combination approaches with low toxicity.

In the current study, we sought to (1) establish a genetically engineered mouse model which recapitulates advanced prostate cancer due to prostate-specific MTA1 overexpression and the loss of PTEN expression (*R26^MTA1^*; *Pten^f/f^*; *Pb-Cre^+^*, hereafter *R26^MTA1^*; *Pten^f/f^*); (2) identify downstream pathways associated with an overexpression of MTA1 in PTEN-loss tumors and examine how these pathways contribute to the cooperative promotion of cancer progression; and (3) investigate the targeted efficacy of gnetin C on MTA1 downstream signaling in vivo.

## 2. Materials and Methods

### 2.1. Reagents and Cell Culture

Gnetin C was a generous gift from Hosoda SHC Co., Ltd. (Fukui, Japan). For in vitro and in vivo experiments, gnetin C was dissolved in dimethyl sulfoxide (DMSO, final concentration 0.1% for in vitro and 10% for in vivo) and stored in the dark at −20 °C until use.

PC3M cells were a gift from Dr. R. Bergman (University of Nebraska Medical Center, Omaha, NE, USA). PC3M cells were cultured in RPMI-1640 media containing 10% fetal bovine serum in an incubator at 37 °C with 5% CO_2_. MTA1 knockdown PC3M cells were generated as described previously [24] using three different shRNAs and selecting clone #3 for demonstrating the most efficient knockdown for the current study (Supplementary Figure S1). Cells were certified as being mycoplasma-free using the Universal Mycoplasma Detection Kit (ATCC, Manassas, VA, USA).

### 2.2. RNA Sequencing

Prostate tissues of 18-week-old mice from the following four genotypes were used for RNA-Seq: (1) *R26^MTA1^*; *Pb-Cre^−^* (WT); (2) *R26^MTA1^*; *Pb-Cre^+^* (MTA1 OE); (3) *Pten^f/f^*; *Pb-Cre^+^* (Pten-null); and (4) *R26^MTA1^*; *Pten^f/f^*; *Pb-Cre^+^* (Pten-null MTA1 OE). Genotypes were confirmed by PCR-based tail genomic DNA genotyping using primers previously described [12]. The total RNA was isolated using the miRNeasy Kit (Qiagen, Hilden, Germany) and sent to LC Sciences (Houston, TX, USA) for RNA sequencing. RNA-Seq libraries were generated from two biologically independent replicates per genotype (described above). The obtained sequencing reads were aligned to the GRCm39 mouse reference transcriptome from the Ensembl genome database (https://www.ensembl.org, accessed on 22 March 2023) and transcript abundance was aggregated to gene-level count using biomaRt (Bioconductor R-package, https://www.bioconductor.org/, accessed on 22 March 2023). Differentially expressed genes (DEGs) among the groups were identified using DESeq2 [25] and were defined using a cutoff of FDR < 0.05 and absolute log_2_ fold change set at 1. To determine the underlying potential signaling pathways enriched among groups, the DEGs were compared with gene sets curated in the Molecular Signatures Database (MSigDB) [26] for the “Hallmark gene sets” collection with FDR < 0.05 for multiple comparisons.

### 2.3. Western Blot Analysis

Protein lysates from cells and prostate tissues were separated using 6%, 10%, or 15% polyacrylamide gels and transferred onto polyvinylidene difluoride membranes as described previously [13]. Membranes were probed with primary antibodies for MTA1, AKT, p-AKT^Ser473^, mTOR, p-mTOR^S2448^, p-S6K^T389^, p-4EBP1, and CyclinD1 from Cell Signaling Technology, Beverly, MA, USA. β-actin and HSP70 were used as loading controls (Santa Cruz Biotechnology, Dallas, TX, USA) (Supplementary Table S1). Signals were detected on ChemiDoc (Bio-Rad, Hercules, CA, USA) using enhanced chemiluminescence (Thermo Fisher Scientific, Somerset, NJ, USA). Densitometry was performed using Image J, v 1.54h (NIH, Bethesda, MD, USA). Quantitation was carried out using the ratio of the intensity of the phosphorylated protein to the total protein of interest versus loading control.

### 2.4. Animals and Study Design

All mice were housed in a pathogen-free facility, and all procedures were performed in accordance with policies and guidelines outlined by the Long Island University Institutional Animal Care and Use Committee (IACUC, approved protocol ID # 2022-011, 10 March 2022). Mice had free access to drinking water and food and were monitored daily for their general health.

*Mouse Models:* Mice with biallelic overexpression of MTA1 in the prostate (*R26^MTA1^*; *Pb-Cre^+^*) have been described previously by us [12]. *R26^MTA1^*; *Pb-Cre^+^* mice then interbred with C57BL/6J mice homozygous for the “floxed” allele of *Pten* gene (*Pten^f/f^*) (Jackson Laboratories, Nashville, TN, USA) to obtain *R26^MTA1^*; *Pten^+/f^*; *Pb-Cre^+^* [12,13] and *R26^MTA1^*; *Pten^f/f^*; *Pb-Cre^+^* male mice (hereafter *R26^MTA1^*; *Pten^f/f^)* for this study. Tail genotyping was performed using the following primers: MTA1 F: 5′-GCT GCT CTC ATC CTC AGA AAC C-3′; MTA1 R: 5′-CTC GAT GTT GTG GCG GAT CTT GAA GTT-3′ with a band of 715 bp; PTEN F: 5′-CAA GCA CTC TGC GAA CTG AG-3′; PTEN R: 5′-AAG TTT TTG AAG GCA AGA TGC-3′ with a wild-type band of 156 bp and mutant band of 328 bp; and Cre F: 5′-TCG CGA TTA TCT TCT ATA TCT TCA G-3′; Cre R: 5′-GCT CGA CCA GTT TAG TTA CCC-3′ with a band of 392 bp [12]. PCR was performed on an Eppendorf thermocycler. *R26^MTA1^*; *Pb-Cre^–^* mice with normal prostates (*n =* 3) served as a reference control group.

Experimental Design: Dose calculations: In our previous studies with transgenic mice and pterostilbene i.p. administration, we found that pterostilbene had anticancer effects at 10 mg/kg bw without any toxicity [10]. Given gnetin C’s improved pharmacokinetic profile [27] and its reported safe dose in animal studies [28], we selected a gnetin C dose of 7 mg/kg bw per day. The dose translation formula used for the determination of equivalent human doses (HEDs) [HED (mg/kg) = Animal dose (mg/kg) × Km ratio, where Km = 0.081 for mice] [29] demonstrated clinical relevance at a 7 mg/kg bw dose. This dose translates to 39.69 mg per day (for an average human male of 70 kg bw), which is within the range of doses shown to be well tolerated and safe in human clinical trials with gnetin C [27,30,31,32].

For gnetin C treatment experiments, two groups of *R26^MTA1^*; *Pten^f/f^* mice were weaned at 3–4 weeks of age and treated with daily i.p. injections of either 10% DMSO vehicle (control, *n =* 7) or gnetin C at a 7 mg/kg bw dose (*n =* 11). The mice received their respective treatment for 5 consecutive days, with 2 days off for a period of 12 weeks. Mice were weighed daily and observed for signs of distress following treatment. At the conclusion of the study, mice were sacrificed in accordance with the approved IACUC protocol. Due to the small size of the mouse prostate, animals from the same treatment group were used either for UGS (prostate, seminal vesicles, and bladder) isolation and fixed in formalin and paraffin-embedded for histology and IHC, or prostate tissue protein extraction stored at −80 °C for Western blot analysis. Blood was collected by cardiac puncture upon sacrifice, and serum samples were stored at −80 °C.

### 2.5. Histology and Immunohistochemistry Analyses of Prostate Tissues

Formalin-fixed, paraffin-embedded sections (Reveal Biosciences, San Diego, CA, USA) were stained with hematoxylin and eosin (H&E) for independent histopathologic examination (GC and CY). The severity of prostatic intraepithelial neoplasia (PIN) and adenocarcinoma was scored by a board-certified veterinary pathologist (CY) using the following system: Score 0: No detectable PIN and prostate carcinoma; Score 1: Only PIN is present; Score 2: Both PIN and adenocarcinoma are present, where adenocarcinoma accounts for less than 50% of the prostate gland; Score 3: Both PIN and adenocarcinoma are present, where adenocarcinoma accounts for more than 50% of the prostate gland. A minimum of five random sections from different locations in the prostate tissues were examined for each mouse. The phenotype was scored based on the analyses of multiple sections per mouse.

Slides were subjected to IHC staining using antibodies for Ki67, MTA1, CD31, cleaved caspase 3 (CC3), p-mTOR, p-SK6, and p-4EBP1 (Supplementary Table S1). Briefly, tissues were deparaffinized, hydrated, and treated to exposed target proteins. Active sites were blocked with serum and endogenous peroxidases were quenched. Tissues were incubated in primary antibody overnight, followed by secondary antibody and avidin–biotin complex (Vectastain ABC Elite Kit, Vector Laboratories, Newark, CA, USA). Stain was developed using the ImmPACT DAB kit (Vector Laboratories). Images were taken using an EVOS XL Core microscope (Thermo Fisher Scientific, Somerset, NJ, USA). The staining intensity of the markers was evaluated and scored by GC. The percentage of positive nuclei was evaluated for Ki67. The total nuclear and cytoplasmic staining intensity was scored according to a four-tier system: 1 (weak staining), 2 (fair staining), 3 (moderate staining), and 4 (strong staining). Analyses of IHC staining were performed using at least five randomly selected areas for each sample in each group using Image J, v 1.54h (NIH, Bethesda, MD, USA) or QuPath v 0.5.0 automated bioimage analysis. Image J was used for cell counting and measuring the CD31 endothelial-stained vessel area in mm^2^. QuPath was used to quantitate the presence and severity of PIN and adenocarcinoma.

### 2.6. ELISA

The levels of IL-2 in mice sera (50 µL samples) were determined by using a commercially available kit (Abcam, Boston, MA, USA) as previously described [12,13]. Briefly, samples and standards in duplicate wells were incubated with the antibody mix and detected by adding 3,3′, 5,5′-tetramethylbenzidine substrate. The reaction was read at 450 nm using a microplate reader (Tecan, Mannedorf, Switzerland). The concentration of IL-2 in samples was calculated from a standard curve.

### 2.7. Statistical Analysis

Statistical analyses were performed using GraphPad Prism v9 software (San Diego, CA, USA). The statistical significance of differences between groups was determined by either Student’s *t*-test or one-way ANOVA as appropriate. Data are shown as mean value ± SEM; *p* < 0.05 was considered statistically significant.

## 3. Results

### 3.1. Prostate-Specific MTA1 Overexpression Makes Pten^f/f^ Mice Progress with Invasive Adenocarcinoma

**Figure 1:**
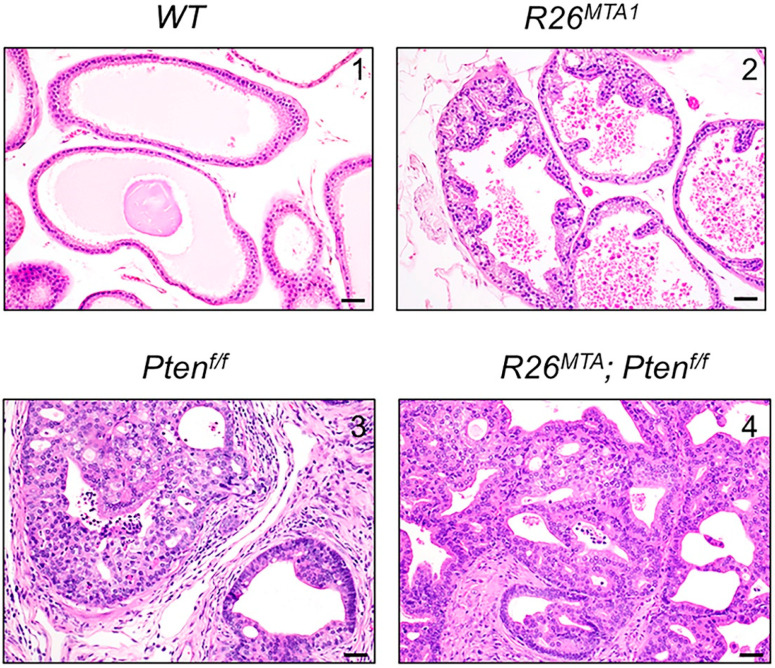
Representative photomicrographs of prostates from four prostate-specific genetic groups: (**1**) *R26^MTA1^*; *Pb-Cre-negative* (WT), (**2**) *R26^MTA1^*, (**3**) *Pten^f/f^*, and (**4**) *R26^MTA1^*; *Pten^f/f^* were evaluated using H&E staining (scale bar, 100 µm). The prostate in WT mice is unremarkable (**upper left**). In *R26^MTA1^* mice, there is an occasional hyperplasia of the prostatic epithelium without nuclear or cellular atypia (**upper right**). In *Pten^f/f^* and *R26^MTA1^*; *Pten^f/f^* mice, both PIN and prostatic adenocarcinoma are observed. The images (**lower left** and **right**) show well to moderately differentiated invasive prostatic adenocarcinoma with desmoplasia and inflammatory infiltrates.

Based on previous studies showing that MTA1 overexpression in the prostate enhances and accelerates PIN development in *Pten^+/f^* mice [12,13], we hypothesized that MTA1 overexpression in the context of PTEN loss-of-function (*Pten^f/f^*) would increase the aggressiveness of prostate cancer progression and might even result in metastasis. Therefore, we generated a prostate-specific *R26^MTA1^*; *Pten^f/f^* mouse model, in which mice develop PIN that progresses to adenocarcinoma. The number of glands exhibiting adenocarcinoma versus PIN in *R26^MTA1^*; *Pten^f/f^* and *Pten^f/f^* mice was comparable. Likewise, the prostate-specific upregulation of MTA1 did not significantly accelerate tumor progression in the context of the loss-of-function of PTEN, and no metastases were found in either renal or iliac lymph nodes of these mice even by 36 weeks of age. Histopathologically, MTA1 overexpression in prostate epithelial cells lacking PTEN expression was characterized by well to moderately differentiated invasive adenocarcinoma composed of neoplastic epithelial cells arranged in glandular structures associated with desmoplasia and variable numbers of inflammatory infiltrates composed of lymphocytes, plasma cells, and neutrophils. There was no evidence of lymphovascular invasion by the neoplastic cells. The neoplastic cells invading the basement membrane did not show a spindle-shaped morphology (Figure 1).

**Figure 2:**
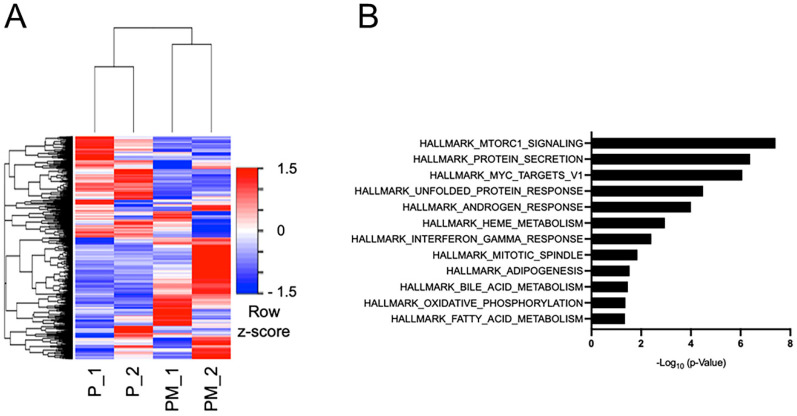
(**A**) Heatmap showing DEGs in the *R26^MTA1^*; *Pten^f/f^* (PM_1 and PM_2) compared to the *Pten^f/f^*; (P_1 and P_2) group. (**B**) Gene set enrichment analysis for the Hallmark gene sets from MSigDB for the DEGs in the *R26^MTA1^*; *Pten^f/f^* compared to the *Pten^f/f^* group.

To identify molecular alterations affected by prostate-specific MTA1 overexpression, we performed RNA-Seq using mouse prostate tissues. A total of 867 genes were found to be significantly upregulated and 1088 genes downregulated (FDR < 0.05) in the *R26^MTA1^; Pten^f/f^* compared to the *Pten^f/f^* group. A heatmap view of the gene clustering of *R26^MTA1^*; *Pten^f/f^* compared to *Pten^f/f^* is shown in Figure 2A (Supplementary Table S2). The pathway most affected by MTA1 overexpression in the PTEN-loss prostate was the mTORC1 (hereafter mTOR) pathway (Figure 2B, Supplementary Table S3), which is known to be aberrantly activated in castration-resistant prostate cancer [33] and advanced prostate cancer with a reduced expression of PTEN [14]. The Principal Component Analysis (PCA) and Multidimensional Scaling (MDS) scatter plot are shown in Supplementary Figure S2. Therefore, our *R26^MTA1^*; *Pten^f/f^* mice mimic a subtype of advanced prostate cancer that exhibits the involvement of mTOR signaling, suggesting that targeting the MTA1/mTOR pathway by natural stilbene gnetin C could be an effective means for blocking prostate cancer progression.

### 3.2. Inhibition of MTA1/mTOR Pathway by Gnetin C in Cell Culture

To gain initial insights regarding the mechanistic basis for targeting the MTA1/mTOR pathway, we first investigated the link between MTA1 and mTOR signaling in PC3M prostate cancer cells, which are known to have a PTEN-null status [34] and the highest MTA1 expression of all prostate cancer cell lines [8]. Oncogenic signaling via the AKT/mTOR pathway is mediated through both mTORC1 and mTORC2, with mTORC1 regulating cell growth by controlling the activity of p70 ribosomal protein S6 kinase (S6K) and eukaryotic translational initiation factor 4E (elF4E)-binding protein 1 (4EBP1), while mTORC2 phosphorylates AKT to promote cell survival [14,35]. Further, the activated mTOR pathway which results in phosphorylated 4EBP1 and S6K leads to higher levels of CyclinD1 [36,37,38], while the inhibition of PI3K/AKT/mTOR/S6K leads to decreased CyclinD1 expression in cancer cells [38,39].

**Figure 3:**
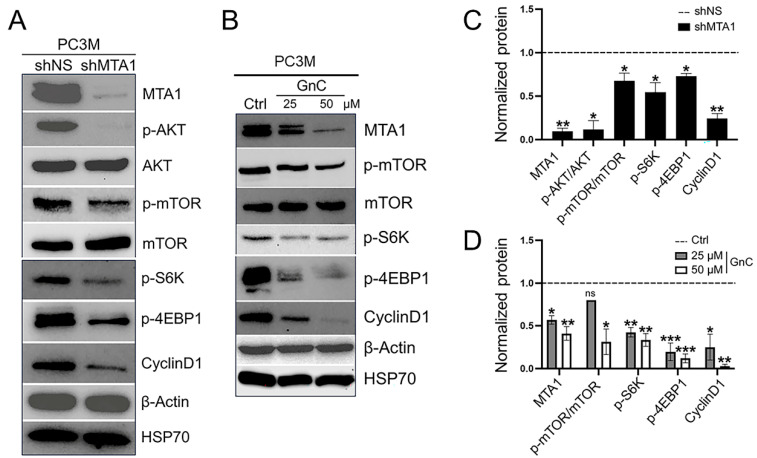
(**A**) Representative Western blot analysis and quantitation of molecular markers of MTA1/mTOR signaling pathway in PC3M MTA1-expressing (shNS) and MTA1 knockdown (shMTA1) cells. (**B**) Gnetin C inhibits MTA1/mTOR pathway markers in a dose-dependent manner. β-actin and HSP70 were used as loading controls. (**C**) Quantitation analysis of molecular markers in PC3M shNS and shMTA1 cells. (**D**) Quantitation analysis of molecular markers in PC3M cells treated with 25 and 50 µM of gnetin C (GnC). Quantitation represents mean ± SEM of three independent experiments. * *p* < 0.05; ** *p* < 0.01; *** *p* < 0.001; ns, non-significant (Student’s *t*-test and one-way ANOVA). The uncropped blots are shown in File S1.

A Western blot analysis of mTOR signaling revealed the marked downregulation of the p-AKT and p-mTOR pathway and its downstream target substrates p-S6K and p-4EBP1 as well as CyclinD1 in PC3M MTA1 knockdown cells compared to control NS cells, indicating that MTA1 acts as an upstream regulator of the mTOR pathway (Figure 3A,C). These data suggest that the MTA1-dependent activation of the mTOR/S6K/4EBP1/CyclinD1 pathway may represent a mechanism through which prostate cancer progresses under conditions of PTEN loss, which suggests a direct positive crosstalk between the MTA1 and mTOR pathway independent of the MTA1/PTEN link [11]. Gnetin C, which previously showed cytotoxicity in PC3M cells with IC_50_ = 8.7 µM [26,27], pharmacologically inhibited MTA1 and reduced the expression of p-mTOR, p-S6K, p-4EBP1, and CyclinD1 (Figure 3B,D) indicating the efficacy of gnetin C for suppressing the MTA1/mTOR pathway.

### 3.3. Gnetin C Treatment Diminishes Adenocarcinoma Progression in R26^MTA1^; Pten^f/f^ Mice

**Figure 4:**
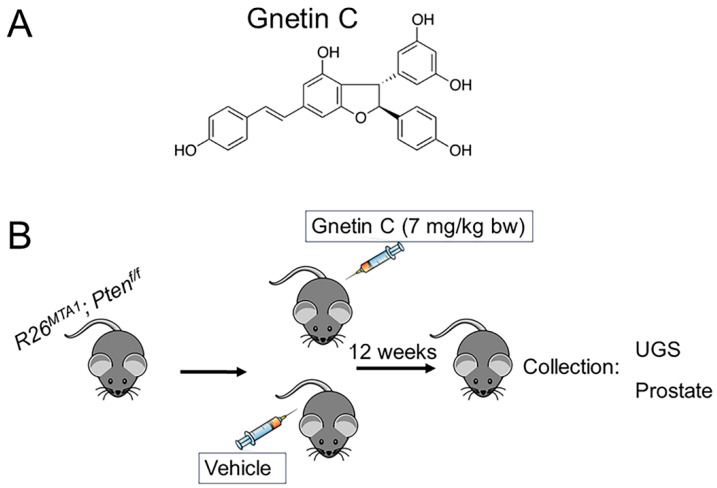
(**A**) Chemical structure of gnetin C. (**B**) Schematic of experimental design used for studying therapeutic effects of gnetin C in advanced prostate cancer murine model. *R26^MTA1^*; *Pten^f/f^* mice were treated with vehicle (*n =* 7) or single daily dose of gnetin C (7 mg/kg bw), (*n =* 11) for 12 weeks, after which UGS or prostate tissues were collected for histopathological and molecular analysis, respectively.

Upon the accumulation of 18 *R26^MTA1^*; *Pten^f/f^* mice, we randomized 4-week-old mice into two groups: mice treated by intraperitoneal (i.p.) injection with vehicle (*n =* 7) or 7 mg/kg bw gnetin C (GnC, *n =* 11) for 12 weeks. Mice were sacrificed in week 13, and prostate tissues and blood were collected for analysis (Figure 4). We also had Cre-negative mice serving as the reference control. Body weight and health condition were monitored. No signs of toxicity were identified.

**Figure 5:**
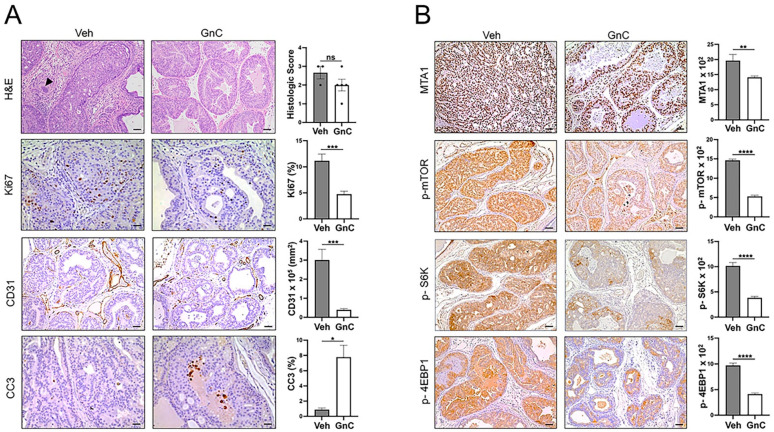
(**A**) **Left**: Representative images of H&E (scale bar, 50 µm). An island of neoplastic epithelial cells invading the adjacent fibrous connective tissue is present (arrowhead). The IHC staining of Ki67, CC3 (scale bar, 20 µm), and CD31 (scale bar, 50 µm) in the prostate tissues from vehicle-treated and gnetin C-treated mice. **Right**: The quantitation of prostate glands involved in PIN vs. adenocarcinoma and the quantitation analysis of Ki67, CD31, and CC3 staining. (**B**) **Left**: Representative images of molecular markers of the MTA1/mTOR pathway in the prostate tissues from vehicle (Veh)-treated and gnetin C (GnC)-treated mice. **Right**: The quantitation of MTA1, p-mTOR, p-S6K, and p-4EBP1 (scale bar, 50 µm) staining. Values are mean ± SEM analyzed from five to seven separate areas per sample (Veh, *n =* 3 and GnC, *n =* 5). * *p* < 0.05; ** *p* < 0.01; *** *p* < 0.001; **** *p* < 0.0001; ns, non-significant (Student’s *t*-test).

Figure 5A (upper panel) shows that in the vehicle-treated group, mice predominantly exhibited adenocarcinoma characterized by the proliferation of the neoplastic glandular epithelium with the invasion of the basement membrane surrounded by desmoplastic reaction and moderate-to-large numbers of infiltrating inflammatory cells composed of neutrophils, lymphocytes, and plasma cells. There is no evidence of lymphovascular invasion by the neoplastic cells. The neoplastic cells invading the basement membrane did not show a spindle-shaped morphology. In contrast, mice treated with gnetin C demonstrated more areas of PIN characterized by the proliferation of glands without the invasion of the basement membrane into surrounding stroma and rare inflammatory cells. The IHC analysis of prostate tissues of mice treated with gnetin C showed a significantly reduced number of Ki67-positive cells and CD31 staining, indicating a significant decrease in proliferation and angiogenesis compared to vehicle-treated mice. Further, we detected increased apoptosis (CC3 staining) in prostates of mice treated with gnetin C compared to vehicle-treated mice (Figure 5A). We next explored the therapeutic potential of gnetin C in inhibiting the MTA1/mTOR pathway. Our finding revealed a significant decrease in MTA1 and the activated mTOR pathway (p-mTOR, p-S6K, and p-4EBP1) in mice treated with gnetin C compared to the vehicle-treated group (Figure 5B).

**Figure 6:**
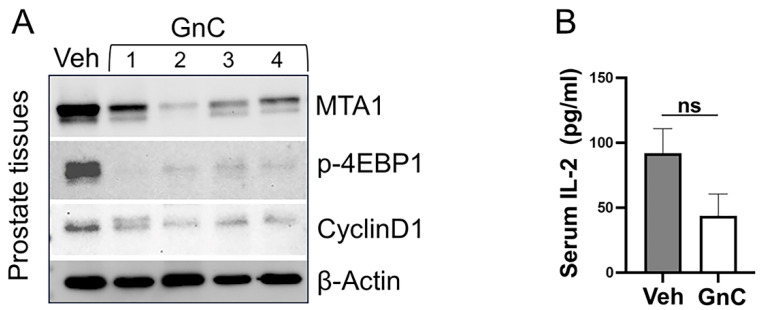
(**A**) Representative immunoblot images of the inhibitory effect of gnetin C (GnC) on molecular markers of the MTA1/mTOR pathway in the prostate tissues from mice in different treatment groups. β-actin was used as a loading control. (**B**) The effect of GnC on circulating IL-2 cytokine levels measured by ELISA in murine sera (*n =* 4 per group). Data represent the mean ± SEM of three independent experiments performed in duplicate. ns, non-significant. Veh, vehicle. The uncropped blots are shown in File S1.

For further analysis, we evaluated the response to treatments by measuring levels of MTA1/mTOR pathway markers in prostate tissue lysates by Western blot (Figure 6A). Although demonstrating noticeable heterogeneity, MTA1 and associated activated mTOR-downstream targets (p-4EBP1 and CyclinD1) levels were significantly downregulated in the prostates of mice treated with gnetin C compared to the vehicle-treated group (Figure 6A). Curiously, the results of p-mTOR and mTOR levels in prostate tissues detected by Western blot were not associated with treatment benefits. This can be explained in part by the very low number of prostate tissues for our Western blot analysis but could also reflect the contradictory role of p-mTOR/mTOR found in prostate cancer patients whose elevated levels positively correlated with a favorable prognosis [40,41]. Existing ambiguity surrounding the biological role of p-mTOR/mTOR in prostate cancer could explain the limited clinical efficacy of PI3K/AKT/mTOR inhibitors [14,40,41]. Nevertheless, the inclusion of MTA1, p-4EBP1, and CyclinD1 in clinical studies may help select patients with high levels of MTA1 that are likely to benefit from gnetin C-targeted therapies.

Finally, we evaluated the anti-inflammatory response to gnetin C treatment in *R26^MTA1^*; *Pten^f/f^* mice by determining the levels of pro-inflammatory IL-2 cytokine in murine sera (Figure 6B). Consistent with our previous observations using low doses of a gnetin C-supplemented diet in a high-risk PIN model of prostate cancer [13], our current study demonstrated that gnetin C was able to reduce the levels of IL-2 compared to the vehicle. 

Taken together, our data demonstrated that gnetin C treatment restored favorable histopathology in *R26^MTA1^*, *Pten^f/f^* mice with a statistically significant reduction in MTA1, accompanied by a reduced rate of cell proliferation and angiogenesis, increased levels of apoptosis, and the inhibition of MTA1-associated markers of activated mTOR signaling, as evidenced by IHC and Western blot analysis. These results suggest that gnetin C has several potent beneficial features, including anti-inflammatory and anticancer properties, defining its therapeutic efficacy in a preclinical mouse model of advanced prostate cancer.

## 4. Discussion

Although mTOR inhibitors are effective in many cancers [42,43,44], they have shown limited efficacy in the treatment of prostate cancer [14,45,46]. In addition, mTOR inhibitor-associated toxicity is a critical problem dictating the need for effective and safe targeted therapies [14,44].

In this study, to discover a novel natural mTOR pathway inhibitor in prostate cancer, we have established a clinically relevant mouse model to test the efficacy of gnetin C-targeted therapy. The prostate-specific *R26^MTA1^*; *Pten^f/f^* genetically engineered mouse model recapitulates features of PTEN-loss advanced prostate cancer facilitated by the overexpression of MTA1.

In our previous studies of MTA1-associated prostate cancer progression, we found an inverse relationship between MTA1 overexpression and PTEN activation, which resulted in an aberrant activation of the AKT signaling pathway [10,11]. In the current study, we have investigated a direct link between the MTA1 and AKT kinase downstream effector, e.g., the mTOR signaling pathway, and found that MTA1 is an upstream regulator of mTOR that is independent of PTEN. Reassuringly, the same association between MTA1 and AKT/mTOR/4EBP1 was recently reported in endometrial cancer, in which miR-30c/MTA1 axes regulated cell proliferation, migration, and invasion via AKT/mTOR signaling defining MTA1 as a therapeutic target in endometrial cancer [47]. Importantly, for the first time, we have pharmacologically targeted the MTA1/mTOR pathway using the natural compound gnetin C both in vitro and in vivo. We report here that gnetin C effectively suppressed the MTA1/mTOR pathway indicated by the downregulated MTA1 and the associated reduced phosphorylation of AKT, mTOR, S6K, 4EBP1, and CyclinD1 in PC3M prostate cancer cells. In prostate tissues, despite the limitations due to a small number of samples, gnetin C showed a significant inhibition of MTA1 and associated p-4EBP1 and CyclinD1. Considering the intricacy of the mTOR signaling network in prostate cancer, it is understandable that preclinical studies would also reflect this complexity. Hence, it becomes important then that there are other biomarkers associated with mTOR that can be indicators of therapy response. Notably, targeting MTA1 by gnetin C consistently resulted in the downregulation of the activated mTOR pathway response markers p-S6K, p-4EBP1, and CyclinD1 detected either by IHC or Western blot analysis. Our data agree with previously reported gnetin C activity on AKT/mTOR signaling in leukemia cells, in which gnetin C’s antitumor effects are demonstrated in cancer cell lines, patient samples, and in vivo through AKT/mTOR and ERK1/2 pathways. This study demonstrated that a combination of gnetin C with low doses of chemotherapeutic drugs leads to a synergistic antitumor effect against acute myeloid leukemia [28].

In the future, modifications of the chemical structure of gnetin C with improved pharmacokinetics could expand therapeutic outcomes when used as monotherapy. Moreover, gnetin C or its derivatives, with their chemosensitizing ability, could be used in combination with clinically approved drugs for synergistic efficacy. We recently demonstrated that gnetin C acts synergistically with enzalutamide to inhibit cell proliferation and angiogenesis and to promote apoptosis in a castrate-resistant xenograft model by the dual-targeting of AR-V7 and MTA1 [48]. While specific MTA1 inhibitors are not available, mTOR inhibitors such as rapamycin and its derivatives are commercially available and suitable for combination therapy in a reduced dose. Therefore, in the future, combinatorial targeted therapies can be beneficial to a biomarker-selected subpopulation of patients with MTA1/mTOR activation-mediated advanced prostate cancer. Further investigations are warranted to determine how MTA1-tumor heterogeneity impacts the effectiveness of treatment and whether target phospho-protein levels can be used as reliable biomarkers for combinatorial treatment response.

## 5. Conclusions

**Figure 7:**
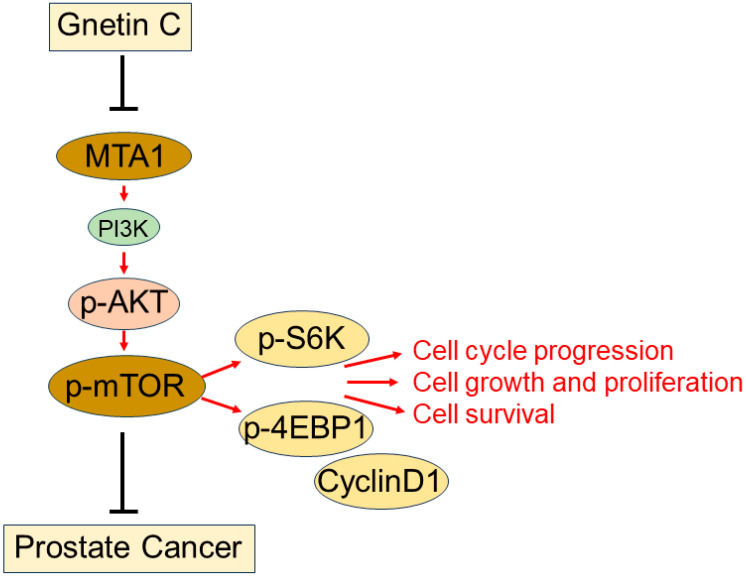
Schematic representation of MTA1/mTOR pathway in prostate cancer. MTA1 overexpression in *R26^MTA1^*; *Pten^f/f^* preclinical model of prostate cancer leads to hyperactivation of mTOR resulting in phosphorylation of downstream target proteins such as S6K and 4EBP1 and CyclinD1. These changes promote cell growth, proliferation, and survival. Gnetin C, plant-derived stilbene, inhibits MTA1-associated mTOR pathway activation resulting in antitumor activity.

We conclude that *R26^MTA1^*; *Pten^f/f^* mice recapitulate key features of MTA1/mTOR signaling pathway activation-associated advanced prostate cancer and represent a useful model for exploring novel therapeutic approaches to manage advanced disease. The discovery of a new mTOR inhibitor with decent efficacy and low toxicity increases the translational potential of the MTA1/mTOR pathway as a therapeutic target. Collectively, our data show that targeting the MTA1/mTOR pathway by the natural stilbene, gnetin C, is highly effective in a preclinical model of prostate cancer and suggests that it could be beneficial for blocking prostate cancer progression in a cohort of patients with a deregulated MTA1/PTEN/AKT/mTOR pathway (Figure 7). Novel natural compounds, such as gnetin C, that target specific biological pathways represent the future for the clinical management of advanced prostate cancer.

## Data Availability

The data generated in this study are available upon request from the corresponding author.

## Supplementary Materials

The following supporting information can be downloaded at: https://www.mdpi.com/article/10.3390/cancers16071344/s1, Figure S1: Validation of MTA1 knockdown in PC3M cells; Figure S2: PCA and MDS scatter plot of gene expression; Table S1: Antibodies used for western blot and IHC; Table S2: Hallmark gene sets; Table S3: Heatmap-PMvP; File S1: Western blot information.

## Abbreviations

AKT	v-akt murine thymoma viral oncogene (protein kinase B)
ANOVA	Analysis of variance
AR	Androgen receptor
bw	Body weight
CC3	Cleaved caspase 3
CD31	Cluster of differentiation 31
CRPC	Castration-resistant prostate cancer
DEGs	Differentially expressed genes
DMSO	Dimethyl sulfoxide
Ctrl	Control
ELISA	Enzyme-linked immunosorbent assay
ERK1/2	Extracellular signal-regulated kinase 1/2
FDR	False discovery rate
GAPDH	Glyceraldehyde 3-phosphate dehydrogenase
f/f	Pten gene flanked by two loxP sites in each allele
GnC	gnetin C
GRCm39	Genome reference consortium mouse build 39
H&E	Hematoxylin and eosin
HED	Human equivalent dose
HSP70	Heat shock protein 70
IACUC	Institutional Animal Care and Use Committee
IHC	Immunohistochemistry
IL-2	Interleukin 2
i.p.	Intraperitoneal
Ki67	Cellular protein marker of proliferation
LIU	Long Island University
mTOR	mammalian target of rapamycin
ns	Non-significant
NS	Non-silenced
OE	Overexpression
p-AKT^S473^	Phosphorylation of serine 473 in C-terminus of AKT
p-4EBP1	Phosphorylated eukaryotic translational initiation factor 4E (elF4E)-binding protein 1
p-S6K	Phosphorylated p70 ribosomal protein S6 kinase
p-mTOR	Phosphorylated mammalian target of rapamycin
Pb-Cre+	Probasin promoter directing expression of epithelial Cre recombinase
PC3M	Human prostate cancer cell line
PCR	Polymerase chain reaction
PI3K	Phosphoinositide 3-kinase
PIN	Prostate intraepithelial neoplasia
PTEN	Phosphatase and tensin homolog
Pten+/f	Pten heterozygous mice
Ptenf/f	Pten homozygous, Pten-null
qRT-PCR	Quantitative reverse transcriptase polymerase chain reaction
RNA	Ribonucleic acid
RPMI-1640	Roswell Park Memorial Institute 1640 cell culture media
R26	Rosa26 loci in the mouse genome
SEM	Standard error of mean
shMTA1	short hairpin MTA1
shNS	short hairpin non-silence
UGS	Urogenital system
WT	Wild-type
