# Supplementary material for: The Therapeutic Efficacy and Mechanism of Action of Gnetin C, a Natural Compound from the Melinjo Plant, in a Preclinical Mouse Model of Advanced Prostate Cancer
Source: Cancers (Basel). 2024 Mar 29;16(7):1344. doi: 10.3390/cancers16071344 (PMC11010822; doi:10.3390/cancers16071344)
Supplement: Supplementary file 1 [file cancers-16-01344-s001.zip › cancers-2893305-File S1.pdf]

Supplementary Information: Original gels

"The Therapeutic Efficacy and Mechanism of Action of Gnetin C, a Natural Compound from the Melinjo Plant, in a Preclinical Mouse Model of Advanced Prostate Cancer"

Gisella Campanelli, Ekniel Francois, Prashanth Parupathi, Lakshmi Sirisha Devarakonda, Ching Yang , Avinash Kumar and Anait S. Levenson

PC3M cells – whole blot images (figure 3a)

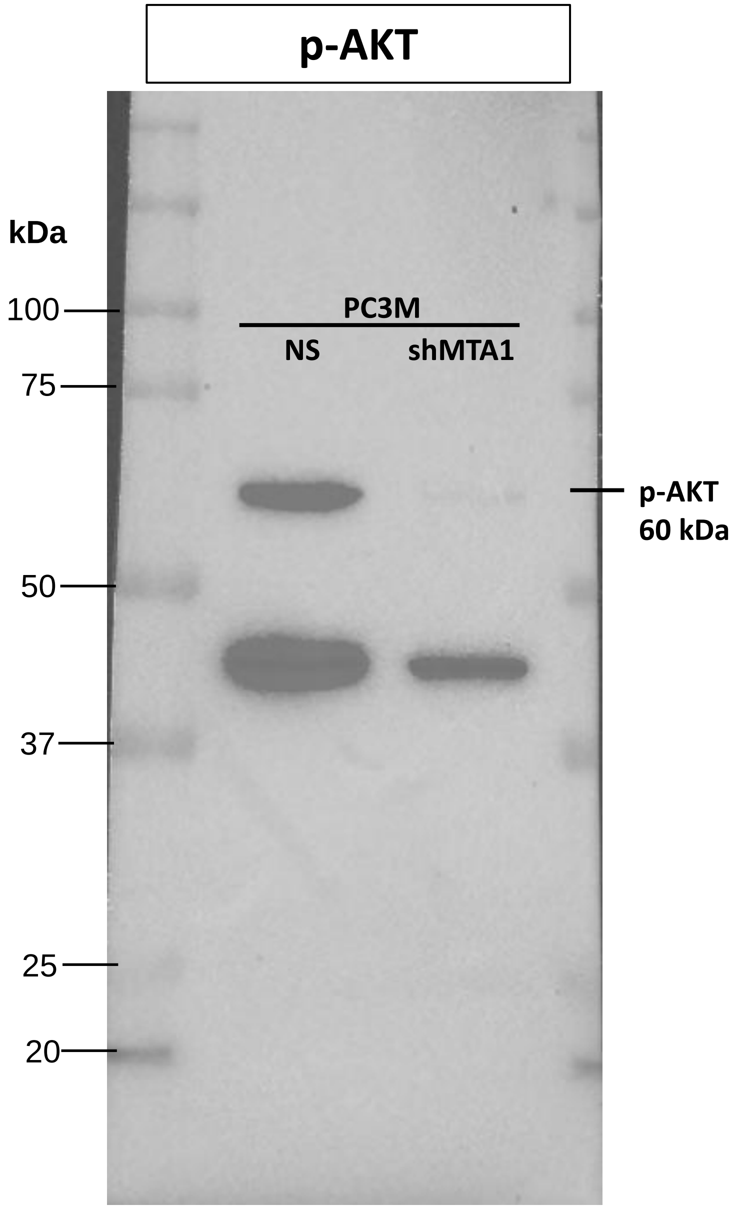

10% polyacrylamide gel

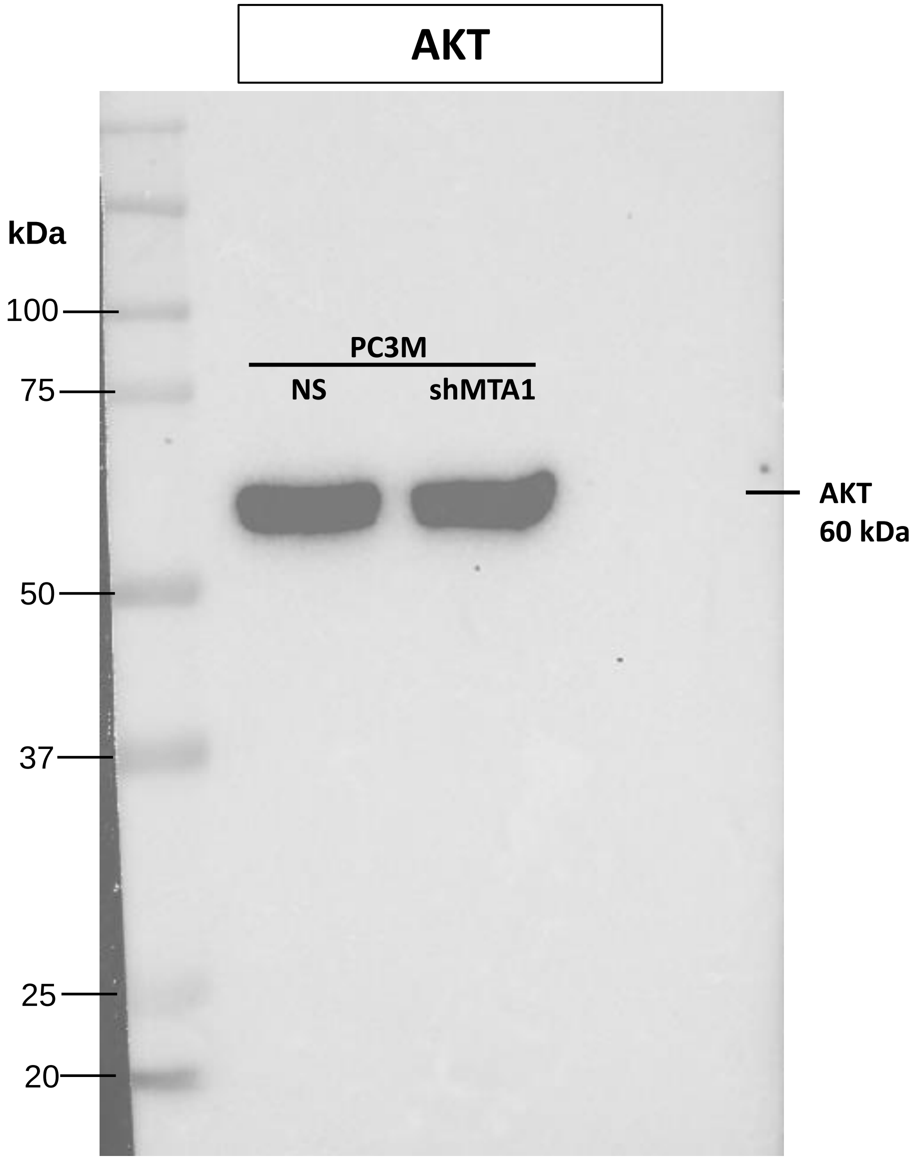

10% polyacrylamide gel

PC3M cells – whole blot images (figure 3a)

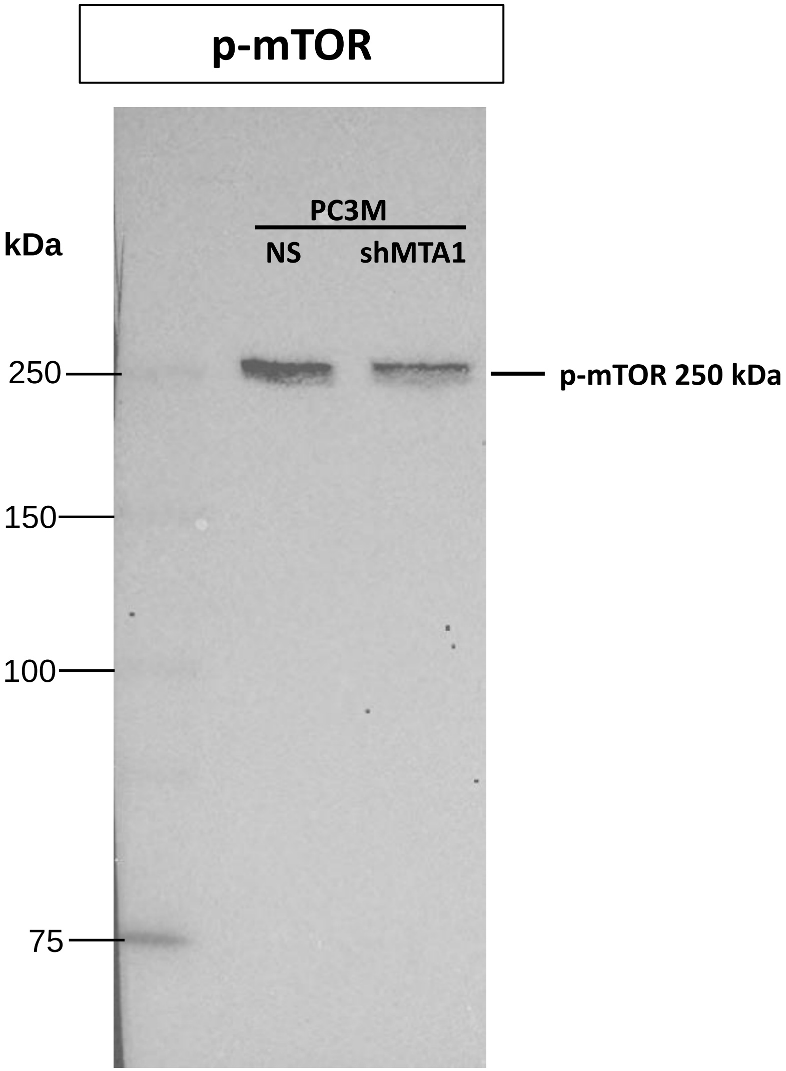

6% polyacrylamide gel

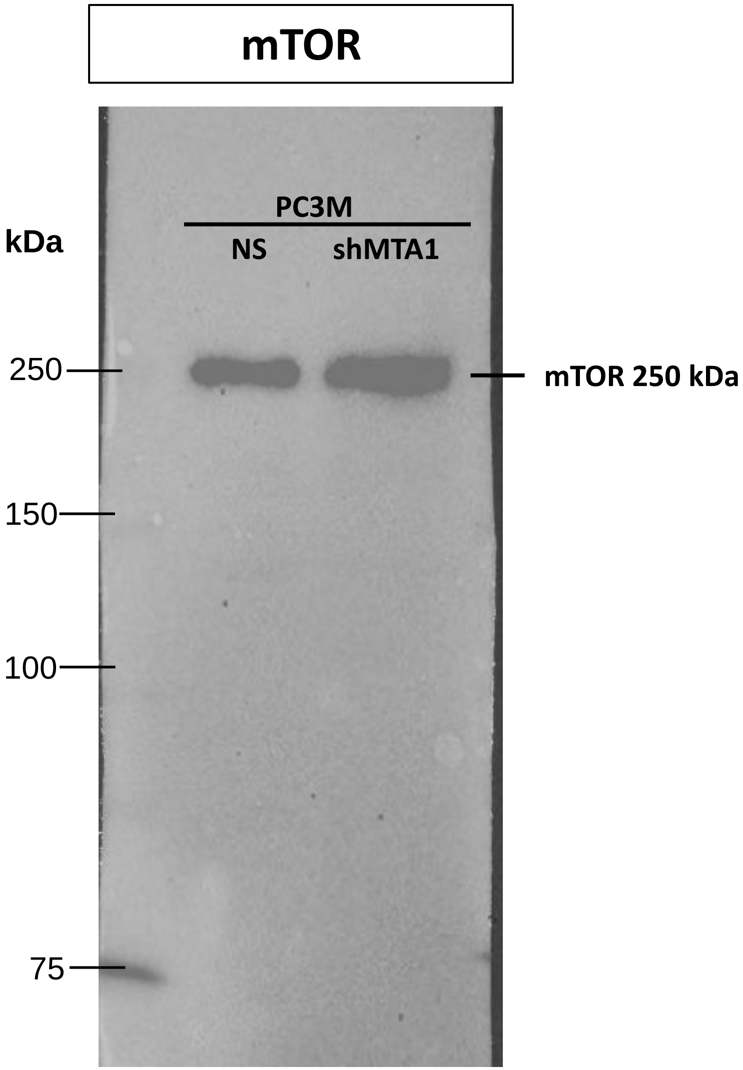

6% polyacrylamide gel

PC3M cells – whole blot images (figure 3a)

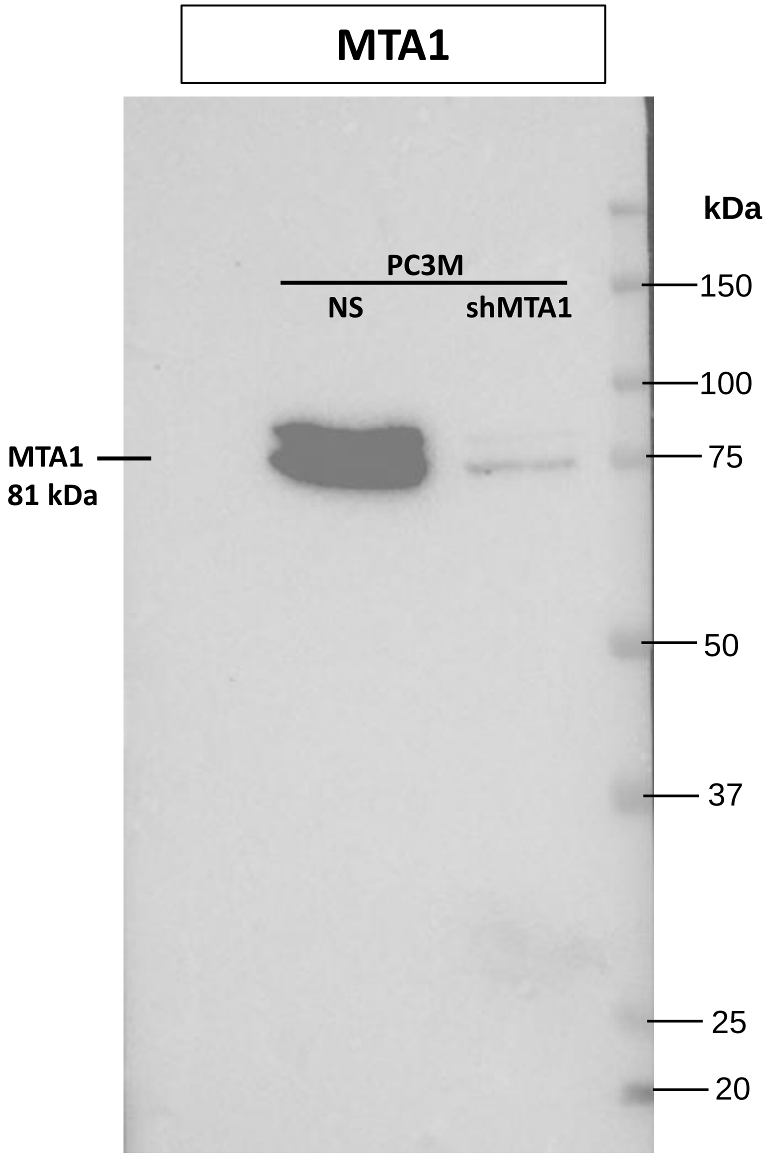

10% polyacrylamide gel

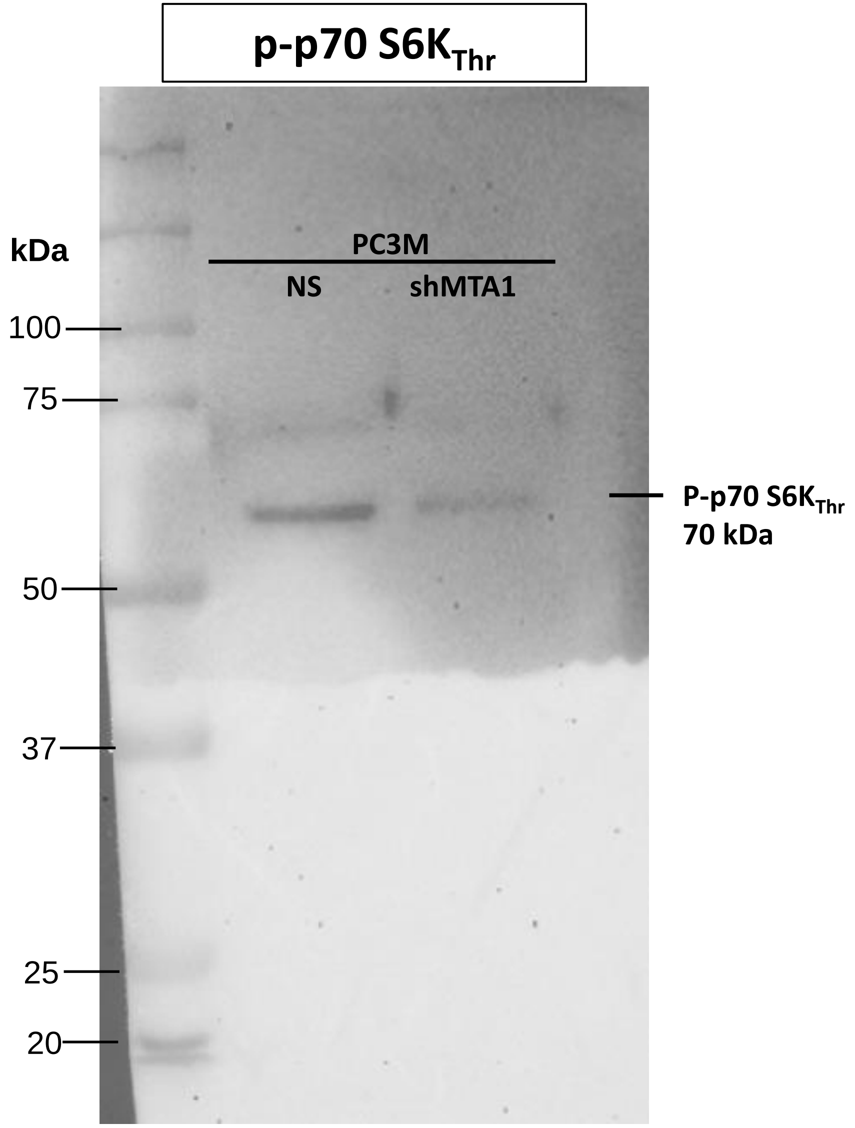

10% polyacrylamide gel

PC3M cells – whole blot images (figure 3a)

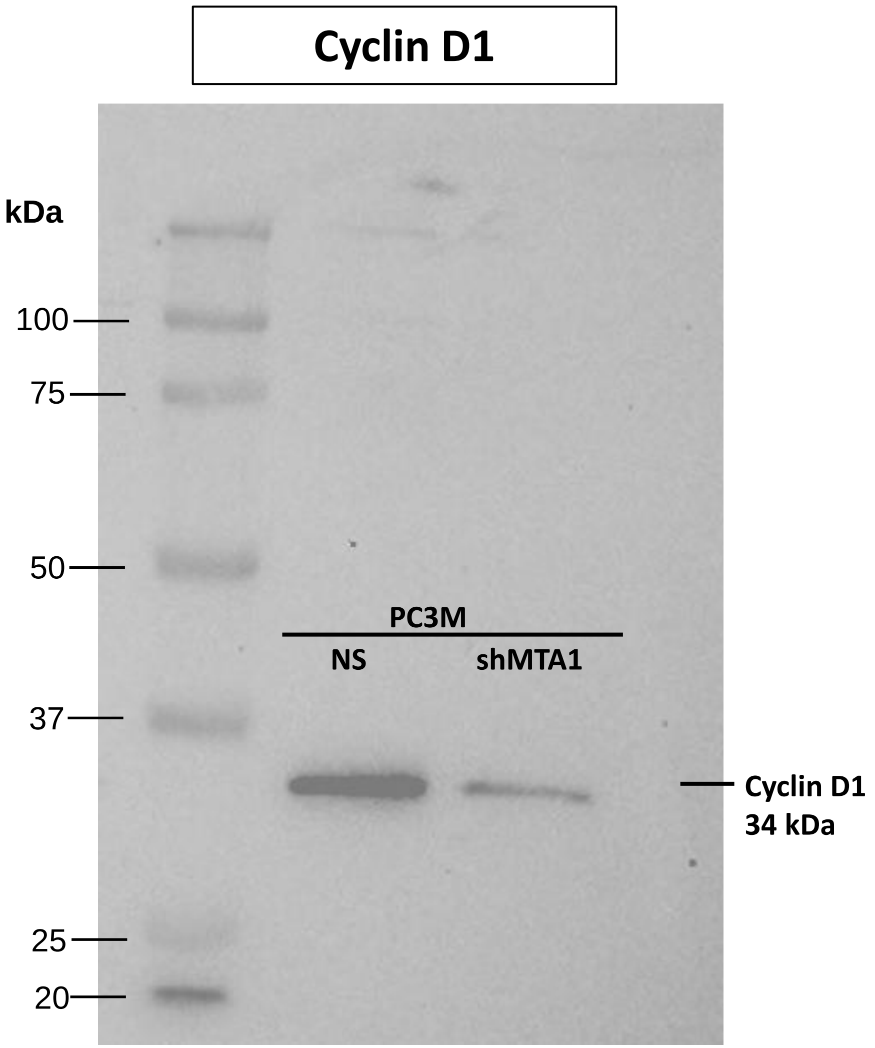

10% polyacrylamide gel

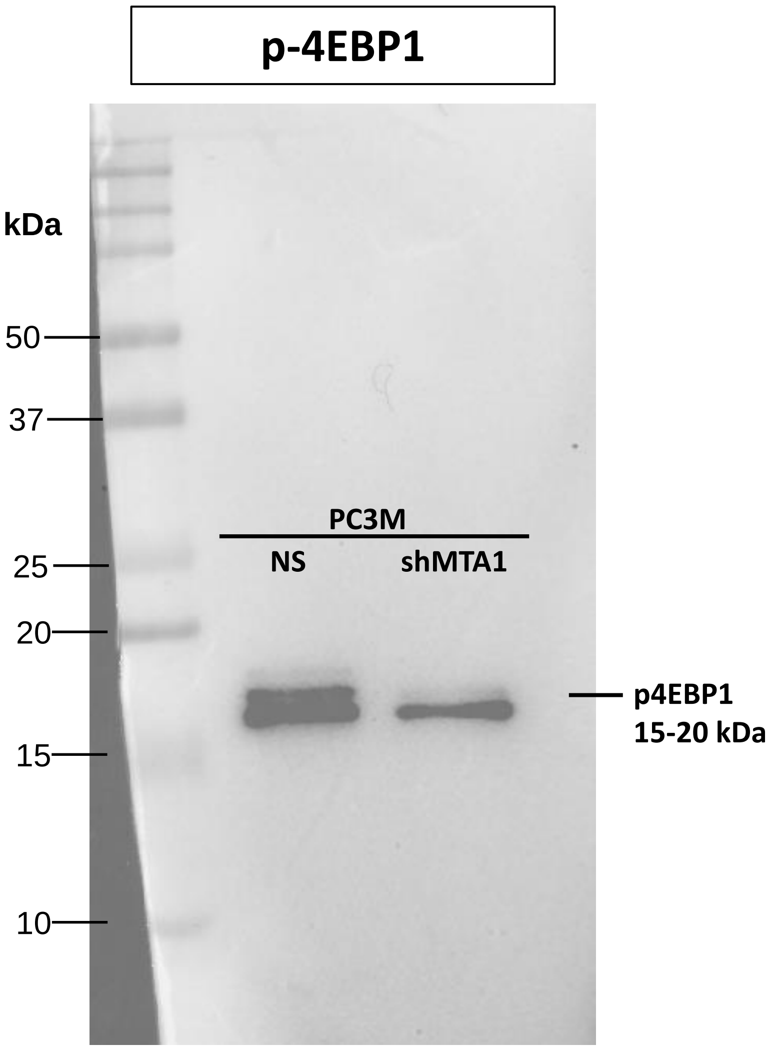

15% polyacrylamide gel

PC3M cells – whole blot images (figure 3a)

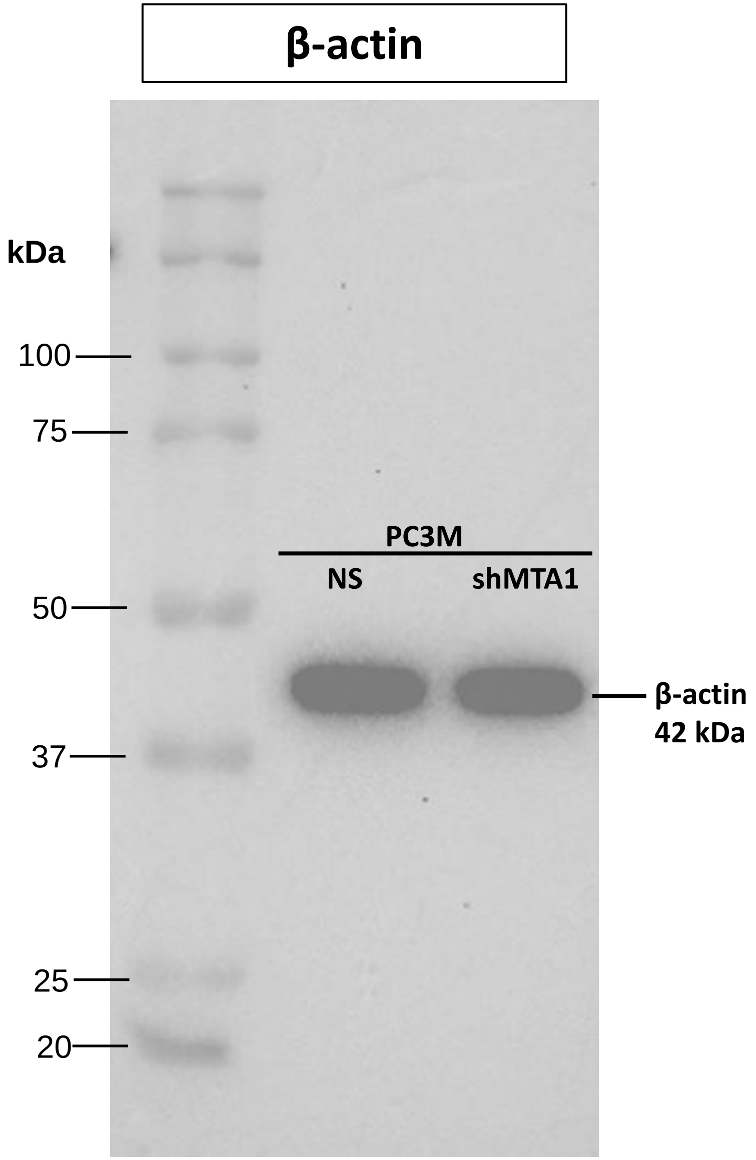

10% polyacrylamide gel

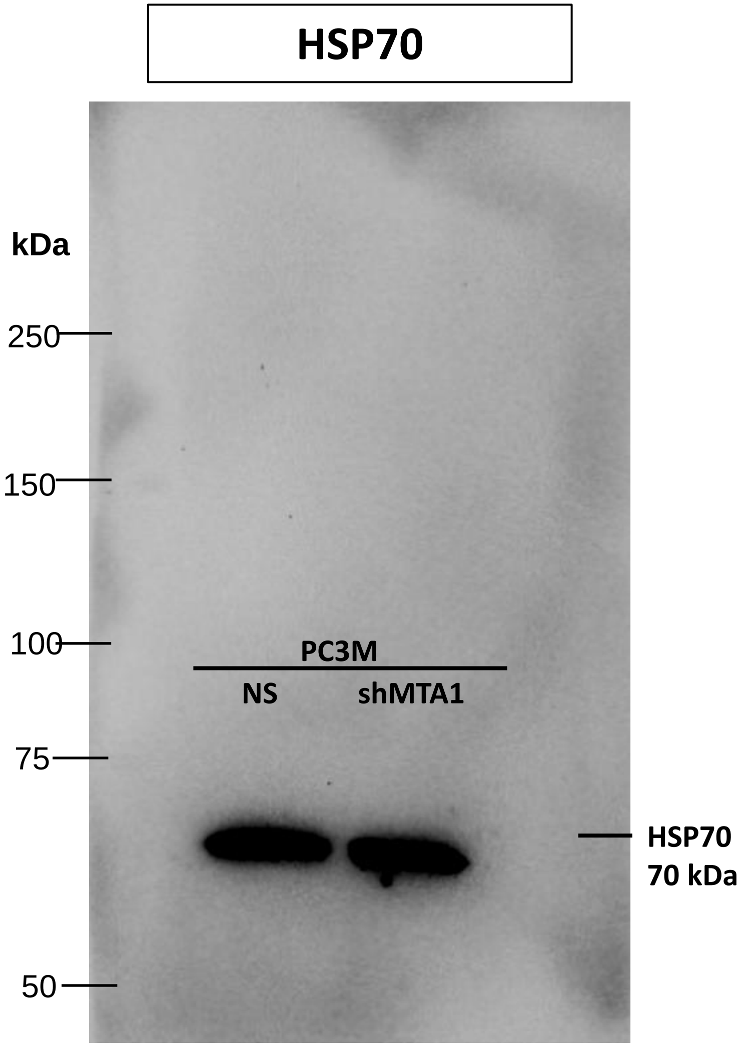

6% polyacrylamide gel

PC3M cells treated with Gnet C – whole blot images (figure 3b)

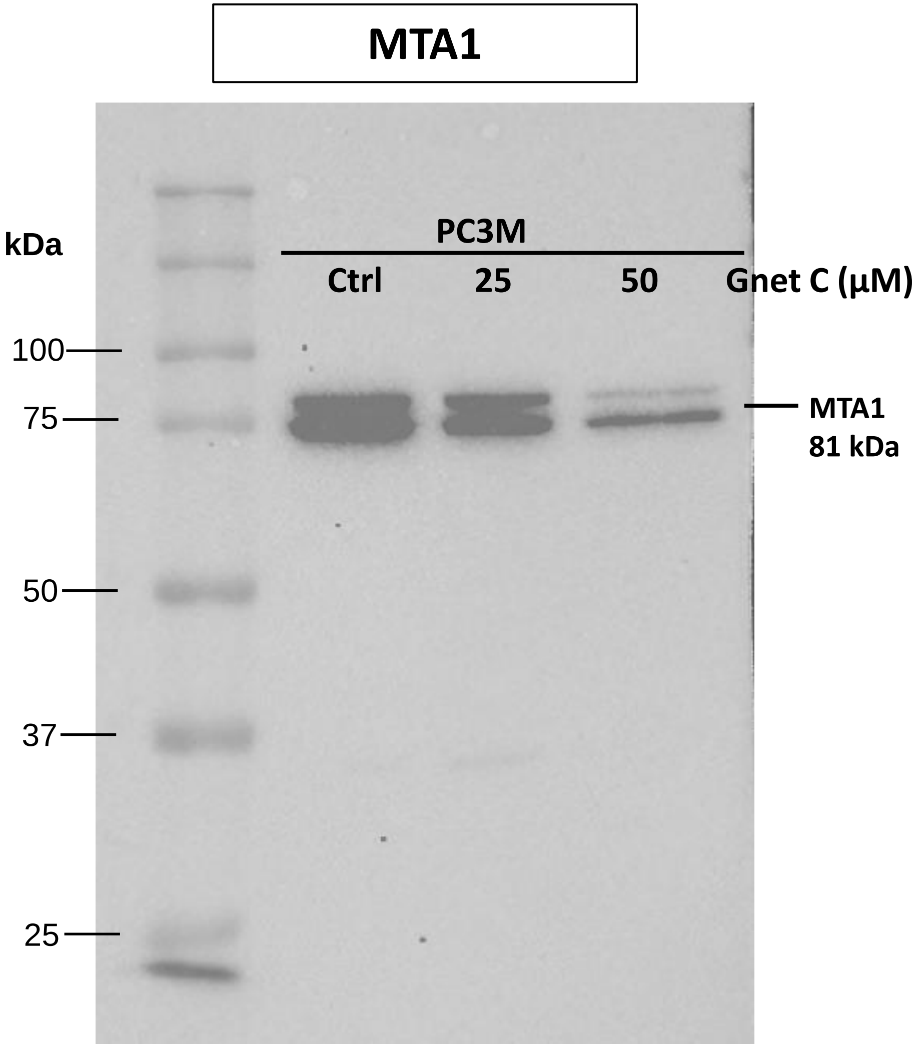

10% polyacrylamide gel

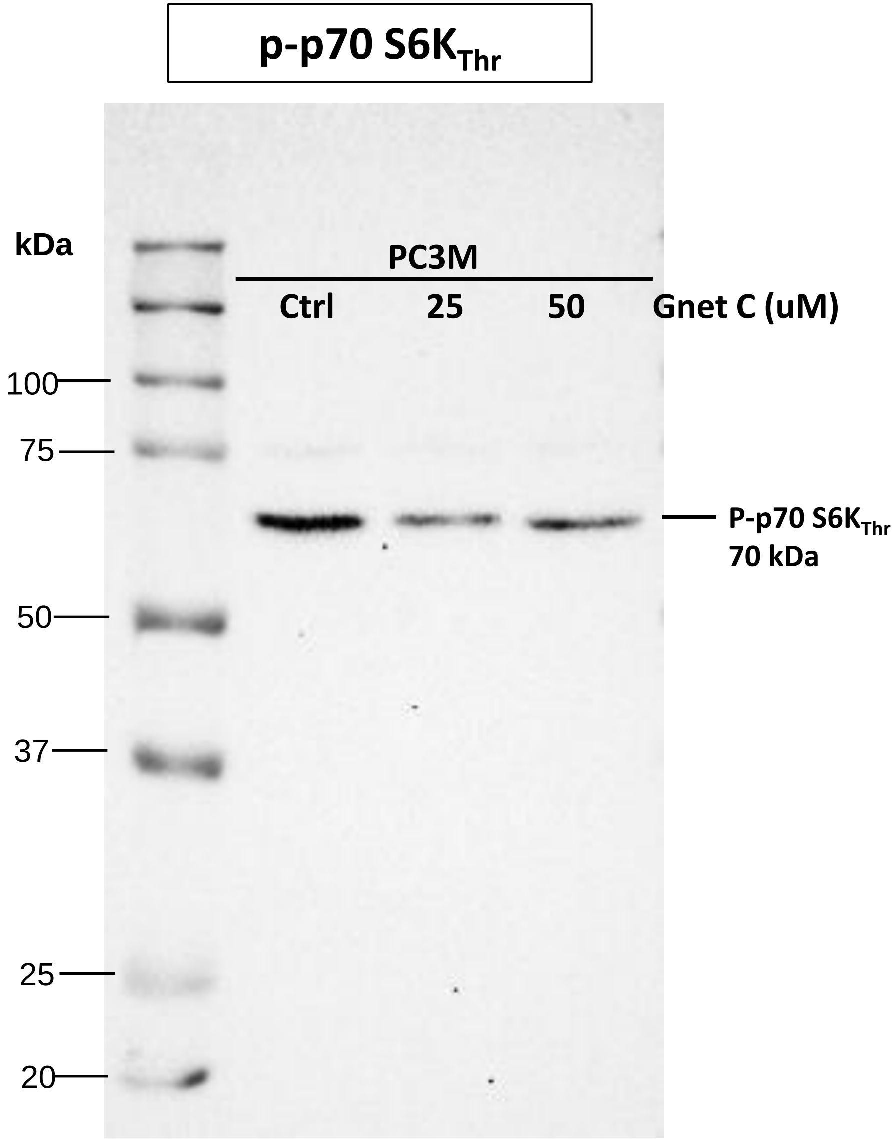

10% polyacrylamide gel

PC3M cells treated with Gnet C – whole blot images (figure 3b)

p-mTOR

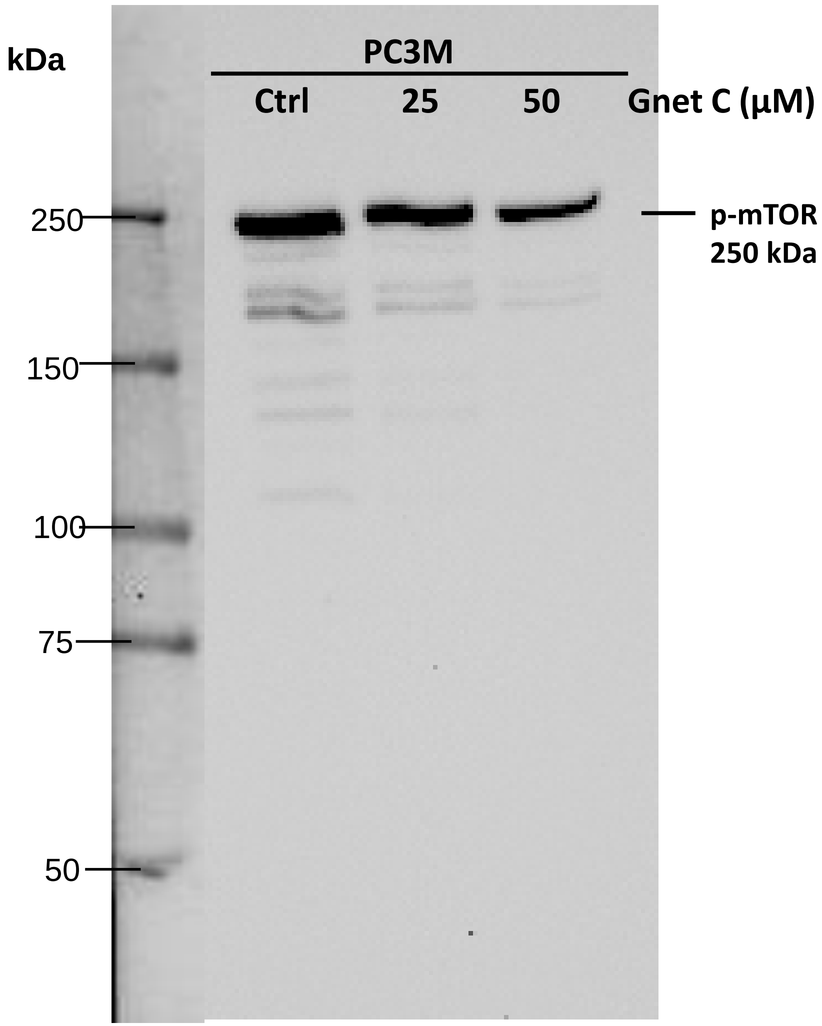

6% polyacrylamide gel

mTOR

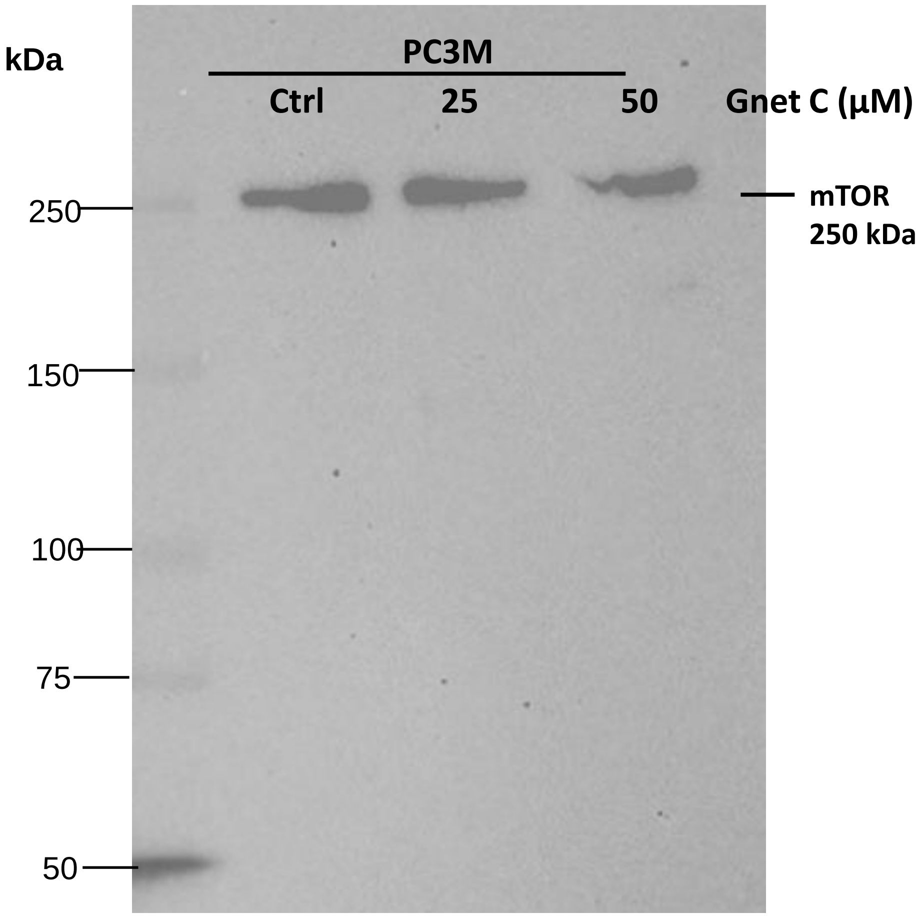

6% polyacrylamide gel

PC3M cells treated with Gnet C – whole blot images (figure 3b)

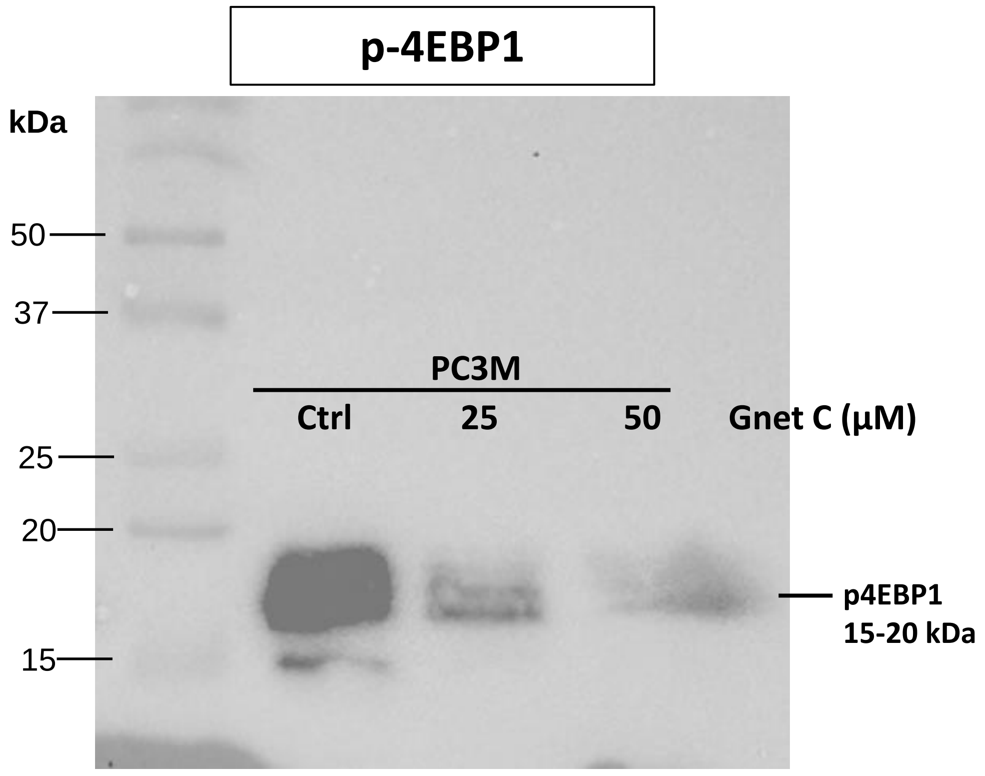

15% polyacrylamide gel

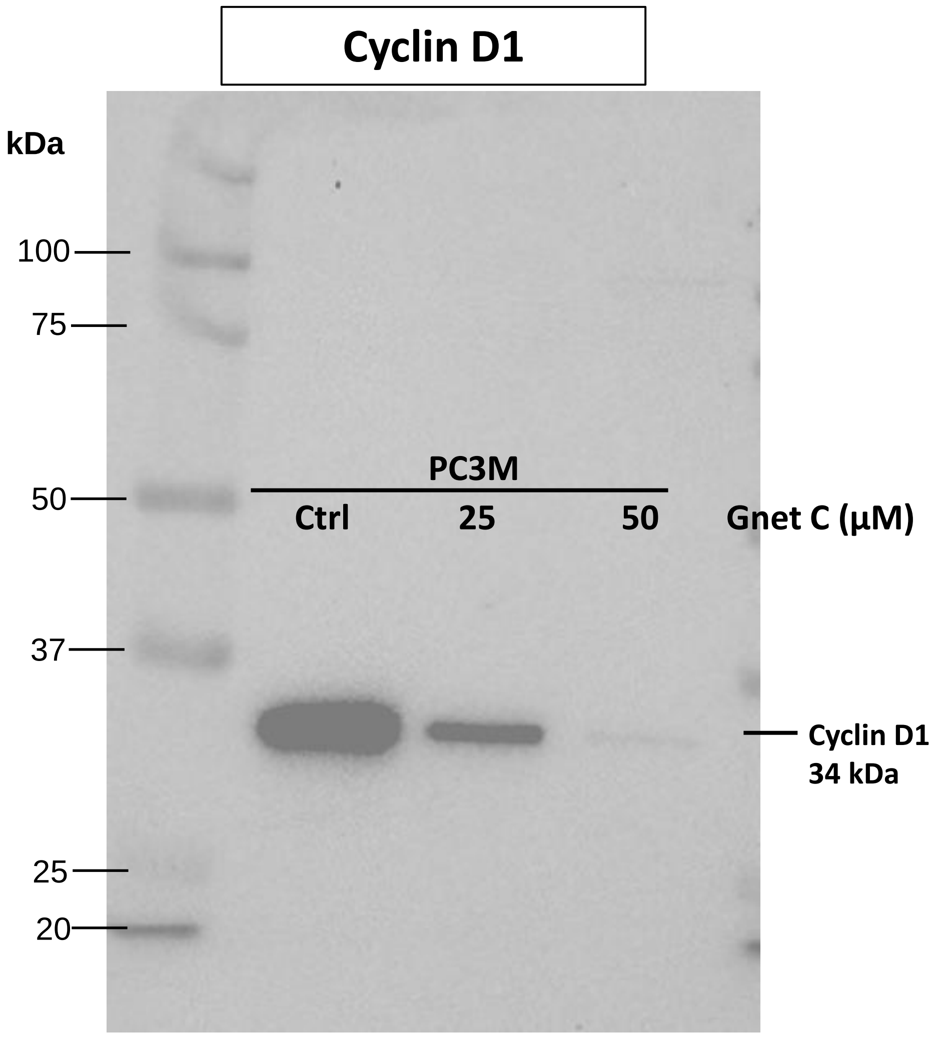

10% polyacrylamide gel

PC3M cells treated with Gnet C – whole blot images (figure 3b)

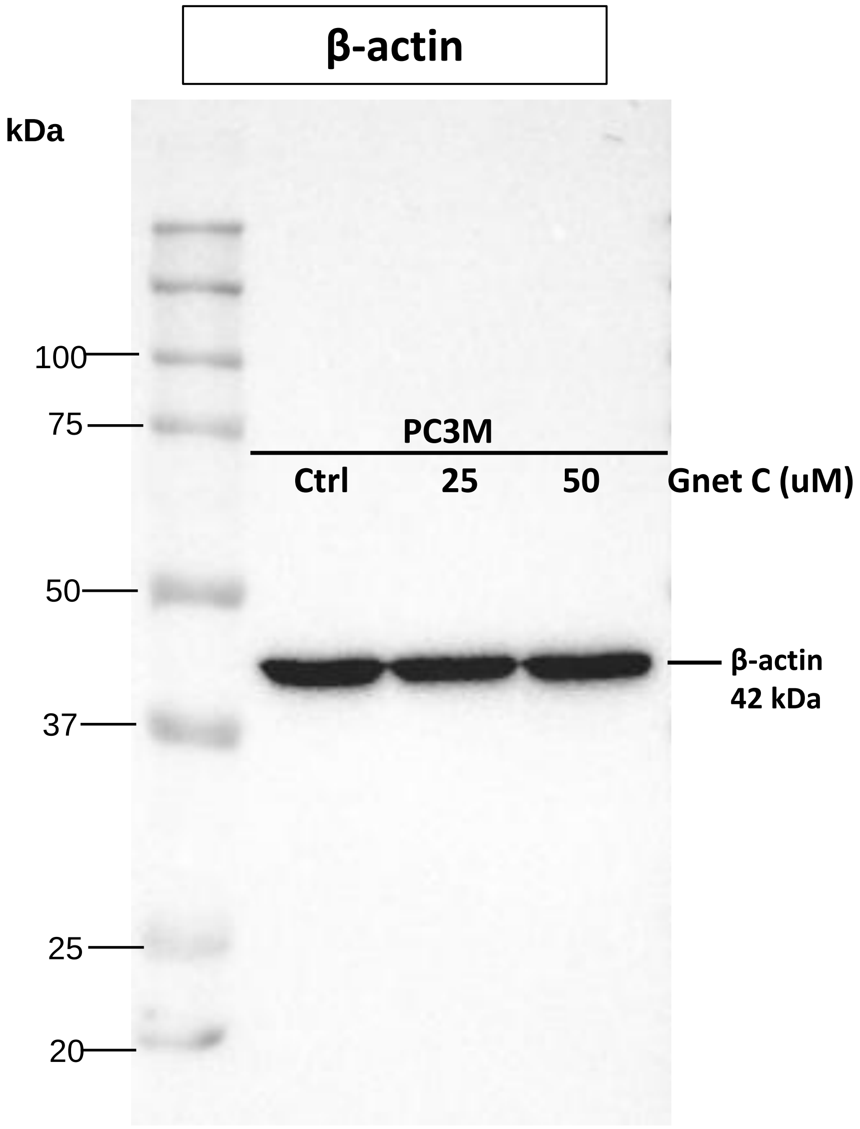

10% polyacrylamide gel

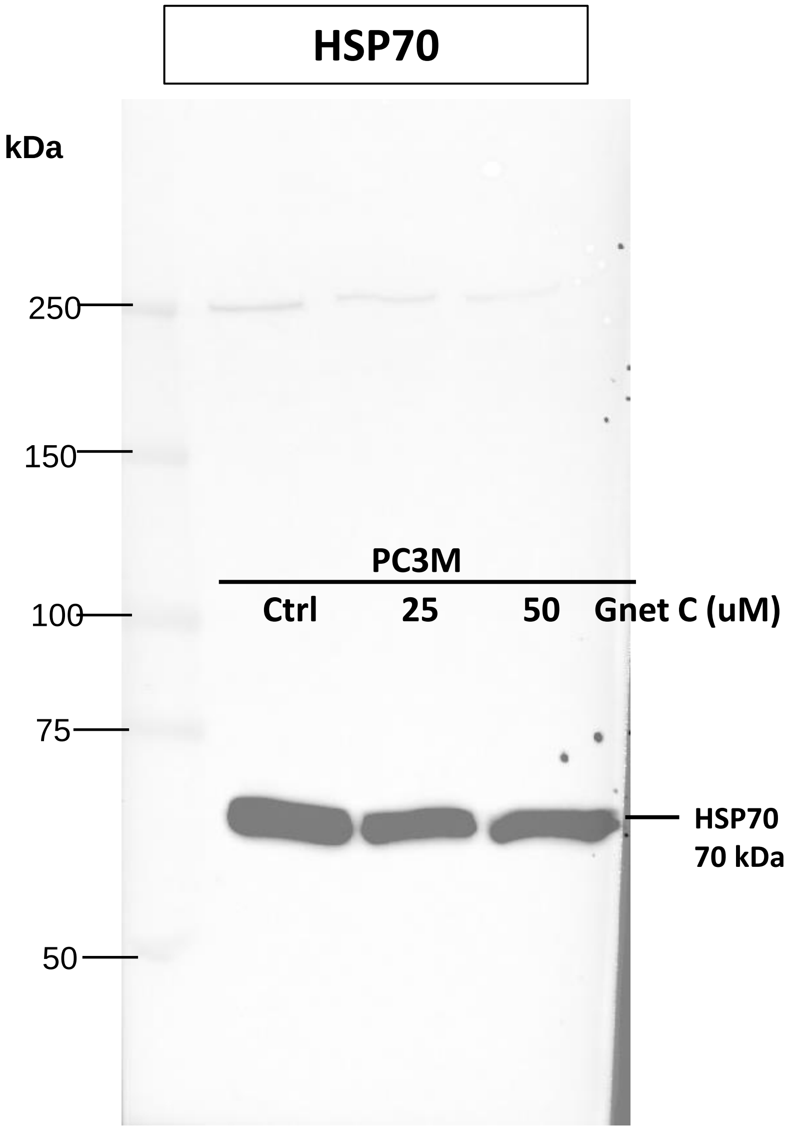

6% polyacrylamide gel

Prostate tissues – whole blot images (figure 6)

MTA1

kDa

100
75
50
37
25
20

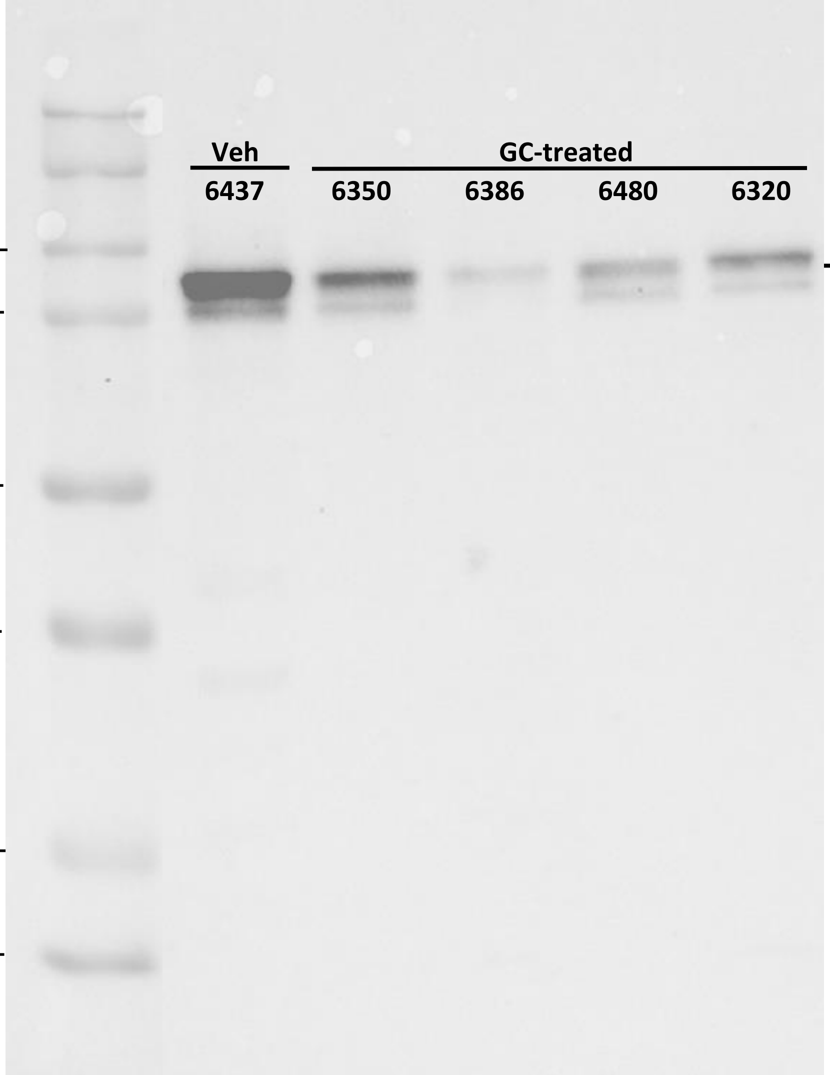

MTA1
81 kDa

10% polyacrylamide gel

p-p70 S6K$_{Thr}$

kDa

Veh
GC-treated
6437
6350
6386
6480
6320

100
75
50
37
25
20

P-p70 S6K$_{Thr}$
70 kDa

10% polyacrylamide gel

Prostate tissues – whole blot images (figure 6)

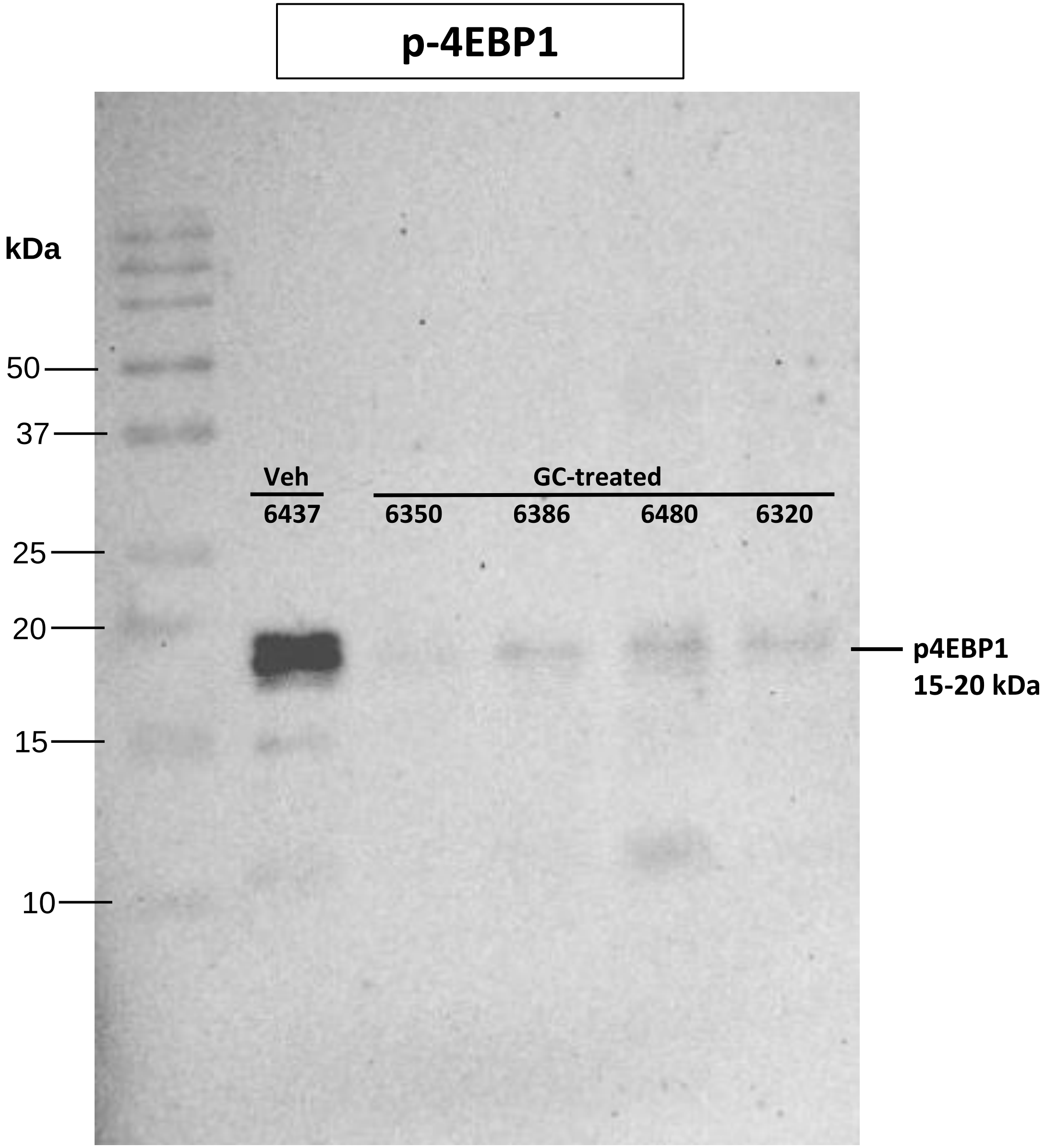

15% polyacrylamide gel

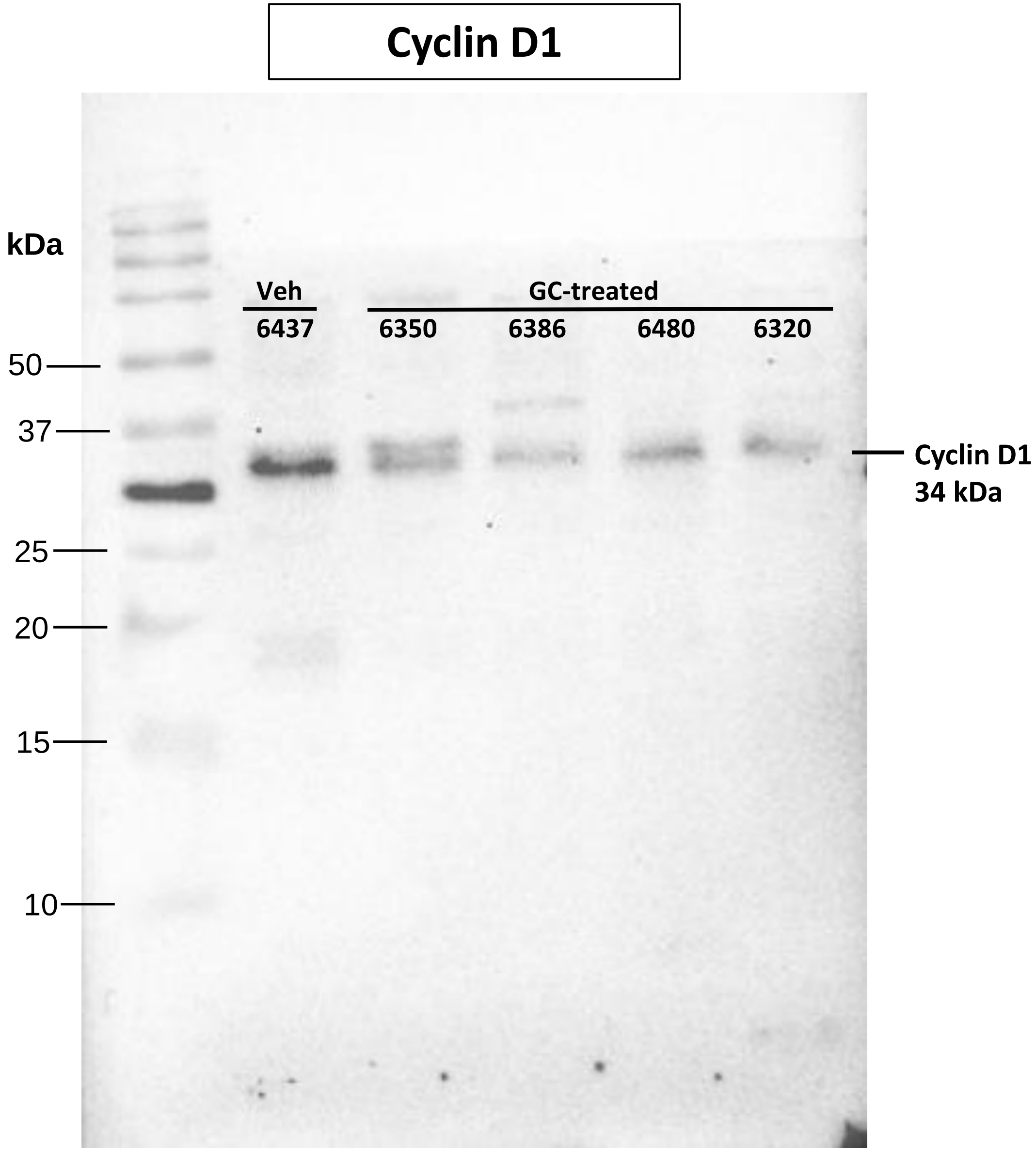

15% polyacrylamide gel

Prostate tissues – whole blot images (figure 6)

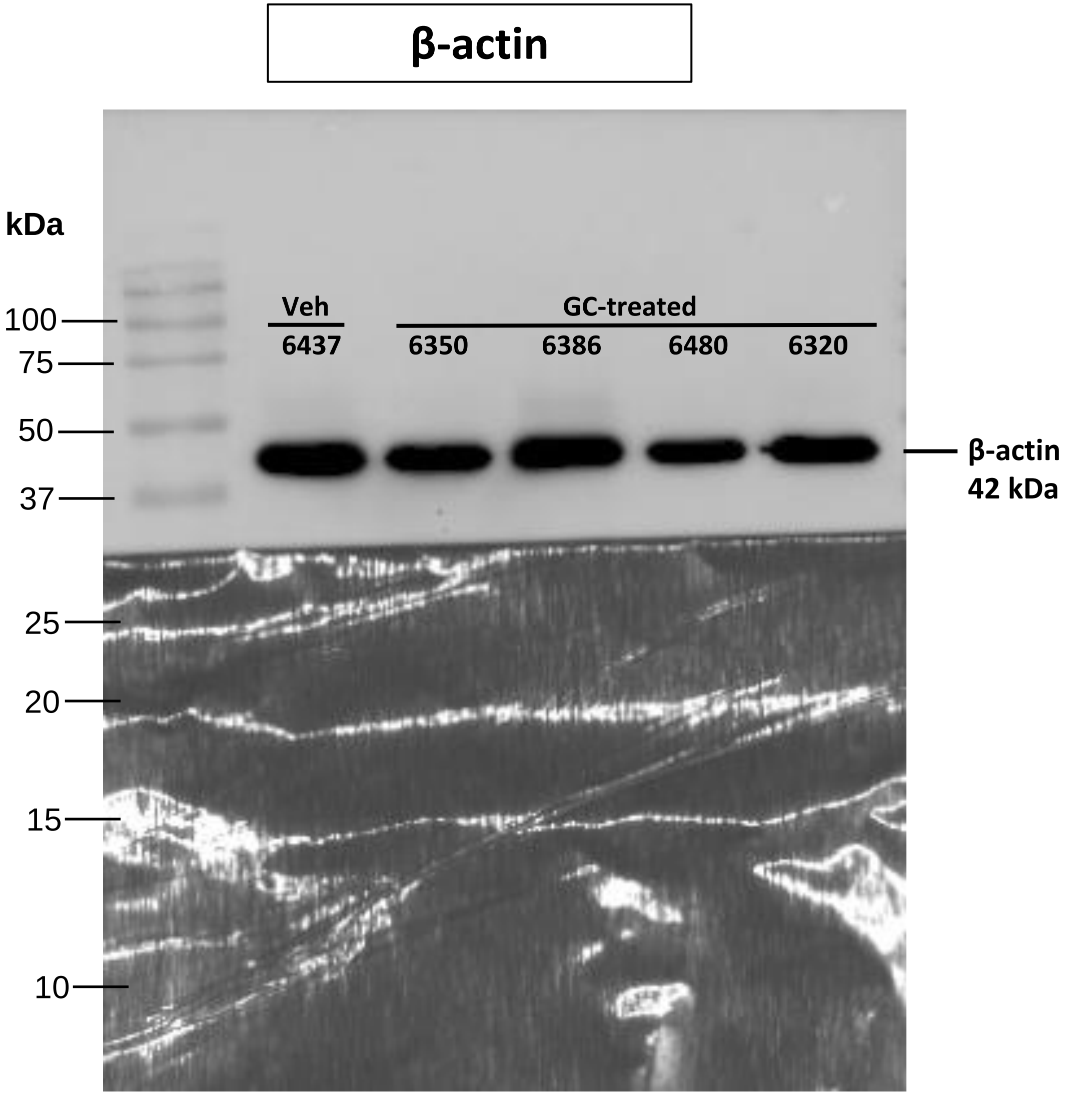

15% polyacrylamide gel
