# Supplementary material for: The Therapeutic Efficacy and Mechanism of Action of Gnetin C, a Natural Compound from the Melinjo Plant, in a Preclinical Mouse Model of Advanced Prostate Cancer
Source: Cancers (Basel). 2024 Mar 29;16(7):1344. doi: 10.3390/cancers16071344 (PMC11010822; doi:10.3390/cancers16071344)
Supplement: Supplementary file 1 [file cancers-16-01344-s001.zip › cancers-2893305-supplementary figures.pdf]

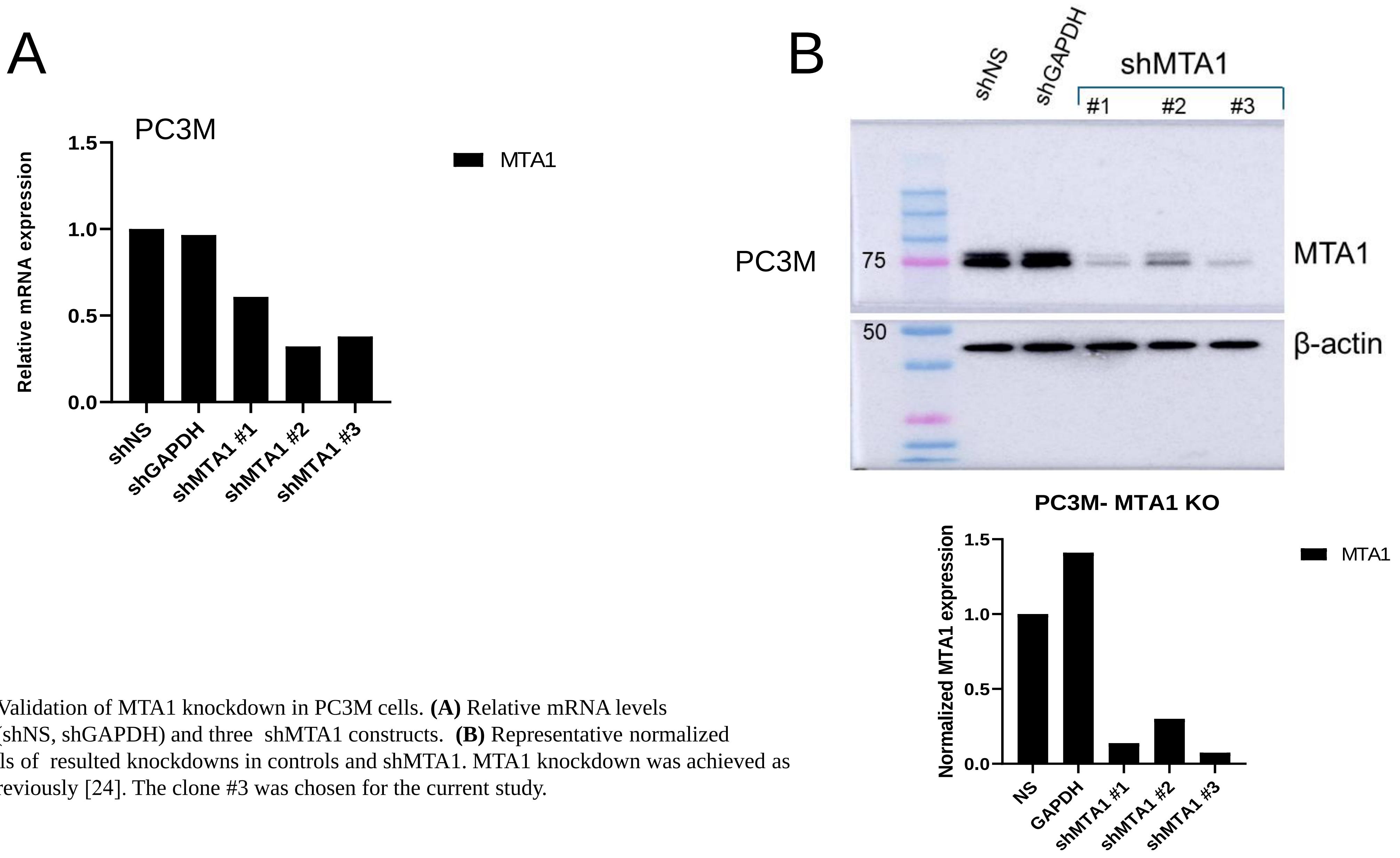

Figure S1. Validation of MTA1 knockdown in PC3M cells. **(A)** Relative mRNA levels of controls (shNS, shGAPDH) and three shMTA1 constructs. **(B)** Representative normalized protein levels of resulted knockdowns in controls and shMTA1. MTA1 knockdown was achieved as described previously [24]. The clone #3 was chosen for the current study.

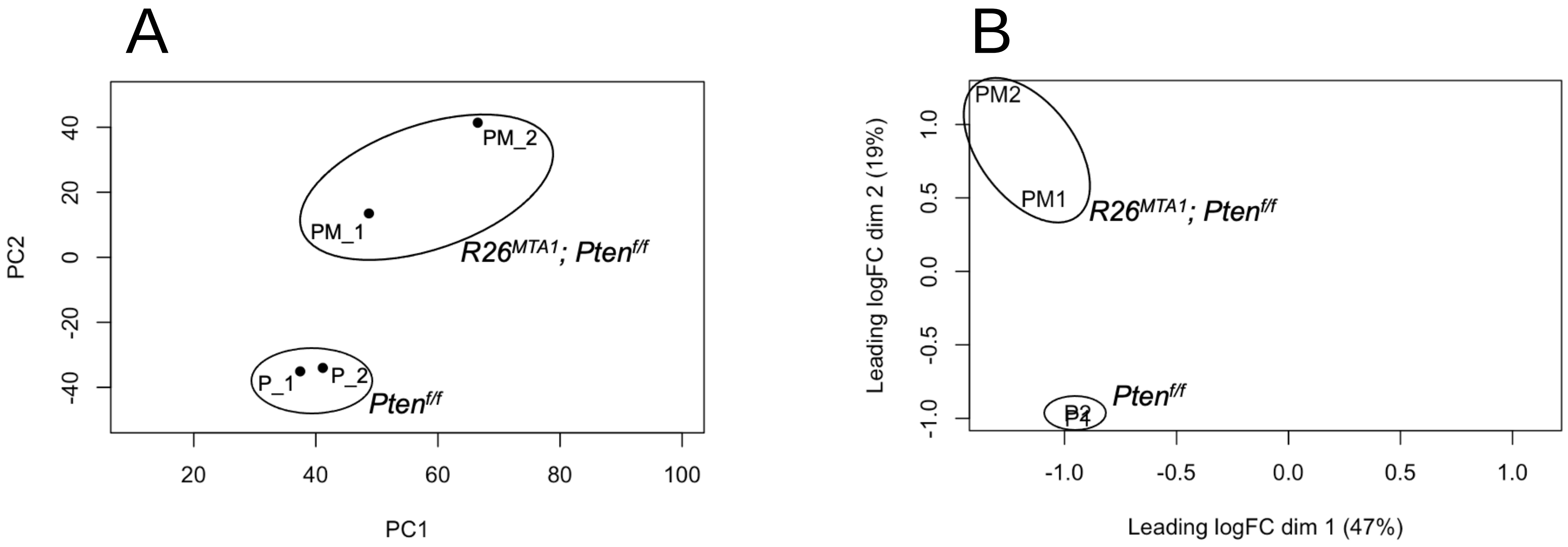

Figure S2. **(A)** Principal Component Analysis (PCA) and **(B)** Multidimensional Scaling (MDS) scatter plot of gene expression showing variance of the two biological replicates of each of the two mouse genotypes, *Pten*$^{f/f}$ and *R26*^{MTA1}*; Pten*$^{f/f}$."
