# Supplementary material for: The Therapeutic Efficacy and Mechanism of Action of Gnetin C, a Natural Compound from the Melinjo Plant, in a Preclinical Mouse Model of Advanced Prostate Cancer
Source: Cancers (Basel). 2024 Mar 29;16(7):1344. doi: 10.3390/cancers16071344 (PMC11010822; doi:10.3390/cancers16071344)
Supplement: Supplementary file 1 [file cancers-16-01344-s001.zip › cancers-2893305-supplementary Table S1.pdf]

SupplementaryMaterial

“The Therapeutic Efficacy and Mechanism of Action of Gnetin C, a Natural Compound from the Melinjo Plant, in a Preclinical Mouse Model of Advanced Prostate Cancer”

Gisella Campanelli, Ekniel Francois, Prashanth Parupathi, Lakshmi Sirisha Devarakonda, Ching Yang , Avinash Kumar and Anait S. Levenson

Table S1. Antibodies used for western blot and IHC.

Antibody	Application	Dilution	Source	Catalog#
AKT	WB (cells)	1:1000	Cell Signaling Technologies	4691
p-AKT (Ser473)	WB (cells)	1:1000	Cell Signaling Technologies	4060
β-actin	WB (tissues)<br>WB (cells)	1:2500<br>1:5000	Santa Cruz Biotechnology	sc-69879
CD31	IHC	1:500	Cell Signaling Technologies	77699
CC3	IHC	1:100	Cell Signaling Technologies	9664
Cyclin D1	WB (tissues)<br>WB (cells)	1:1000<br>1:1000	Cell Signaling Technologies	2922
HSP70	WB (cells)	1:1000	Santa Cruz Biotechnology	sc-24
Ki67	IHC	1:200	Abcam	Ab16667
MTA1	WB (tissues)<br>WB (cells)<br>IHC	1:1000<br>1:2500<br>1:50	Cell Signaling Technologies	5647
mTOR	WB (cells)	1:1000	Cell Signaling Technologies	2983
p-mTOR (Ser2448)	WB (cells)	1:1000	Cell Signaling Technologies	5536
p-mTOR (Ser2448)	IHC	1:100	Santa Cruz Biotechnology	sc-293133
p-4EBP1 (Thr37/46)	WB (tissues)<br>WB (cells)<br>IHC	1:1000<br>1:1000<br>1:1000	Cell Signaling Technologies	2855
P-p70 S6 kinase (Thr389)	WB (cells)	1:1000	Cell Signaling Technologies	9234
P-p70 S6 kinase (Ser371)	IHC	1:50	Invitrogen	PA5-104841
